# Maternal and perinatal outcomes during successive and overlapping crises in Ukraine, 2019–2024: a nationwide population-based ecological study

**DOI:** 10.1016/j.lanepe.2026.101774

**Published:** 2026-07-14

**Authors:** Iryna Mogilevkina, Valeriia Marichereda, Natalia Khadginova, David Southall, Dmytro Dobryanskyy

**Affiliations:** a Institute of Postgraduate Education, Bogomolets National Medical University, Beresteiskyi Avenue, 34, Kyiv, 03057, Ukraine; b Department of Women's and Children's Health, Uppsala University, P.O. Box 256, Uppsala, SE-75105, Sweden; c Department of Obstetrics and Gynaecology, Odesa National Medical University, Valikhovskiy Lane, 2, Odesa, 65082, Ukraine; d State Institution Ukrainian Centre of Maternity and Childhood of the National Academy of Medical Sciences of Ukraine, Platona Mayborody Str., Kyiv, 04050, Ukraine; e Maternal and Childhealth Advocacy International, 1 Columba Court, Laide, Highland IV22 2NL, Scotland; f Department of Paediatrics and Medical Genetics, Danylo Halytsky Lviv National Medical University, Lviv, 79010, Ukraine

**Keywords:** COVID-19 pandemic, War, Ukraine, Maternal morbidity, Pregnancy-related mortality, Perinatal mortality

## Abstract

**Background:**

Ukraine has experienced overlapping shocks from the COVID-19 pandemic and a full-scale war, with potential impacts on maternal and perinatal health. We examined national-level changes in these outcomes between 2019 and 2024.

**Methods:**

In this population-based ecological study, publicly available Ministry of Health data were analysed to examine trends in deliveries, births, and maternal and perinatal outcomes. Changes were assessed using pairwise comparisons, absolute risk differences, and effect sizes. Annual estimates were compared with pre-pandemic, preceding-year, and wartime estimates.

**Findings:**

Deliveries and births declined by approximately 40%, predominantly during the first wartime year. Between 2019 and 2024 (298,066 and 176,842 deliveries, respectively), the prevalence of diabetes in pregnancy increased from 0.88% (n = 2634) to 2.66% (n = 4707), hypertensive disorders from 3.80% (n = 11,332) to 5.45% (n = 9633), severe postpartum haemorrhage from 0.36% (n = 1070) to 0.53% (n = 941). Among 302,190 and 179,192 births, respectively, preterm births increased from 5.59% (n = 16,907) to 6.25% (n = 11,195), very low birthweights from 1.05% (n = 3184) to 1.29% (n = 2305), and extremely low birthweights from 0.44% (n = 1335) to 0.60% (n = 1068). Pregnancy-related and perinatal mortality, particularly stillbirths, were higher during the second year of the pandemic but not during wartime. Early neonatal mortality did not change throughout.

**Interpretation:**

Overlapping crises were associated with fewer births and increased maternal morbidity. Although pregnancy-related and perinatal mortality increased during the pandemic, their relative stability during wartime may suggest preservation of maternal and newborn services, potentially supported by international assistance. Continued support for maternal and neonatal healthcare services remains important in crisis-affected settings.

**Funding:**

No funding was received.

## Graphical abstract

**Figure:**
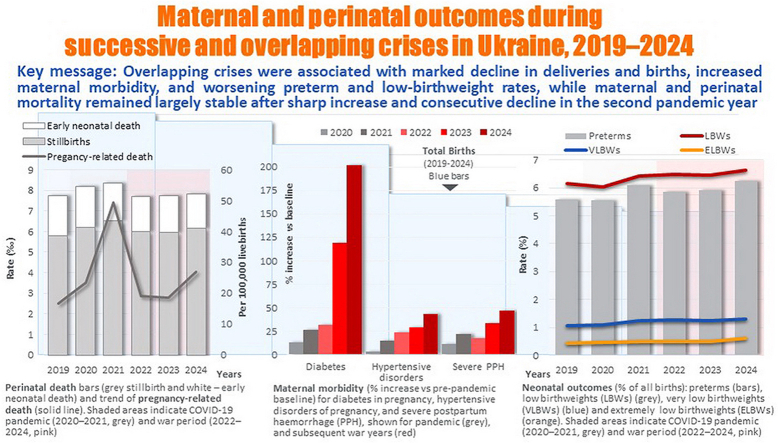

**Research in context**

**Evidence before this study**

To situate our study and assess the existing evidence gap, we conducted a targeted literature search in PubMed and Google Scholar from database inception to Nov 1, 2025, for studies examining the effect of the COVID-19 pandemic and armed conflict on maternal and perinatal health. We used combinations of terms including “COVID 19”, “war”, “conflict”, “war in Ukraine”, “maternal outcomes”, “perinatal outcomes”, “birth outcomes”, and “health system disruption”. We included publications in English and Ukrainian. The search confirmed that both the COVID-19 pandemic and war had disrupted health systems, and access to maternal and perinatal care, and were associated with adverse maternal and perinatal outcomes. Notably, we identified no national-level studies from a European conflict setting that systematically quantified maternal and perinatal health indicators during overlapping crises in which a full-scale war coincided with the COVID-19 pandemic. This evidence gap provided the rationale for the present study.

**Added value of this study**

The study provides the first large-scale quantification of maternal and perinatal health indicators during overlapping crises when a full-scale war within the same population followed the COVID-19 pandemic. Using routine aggregated data, we observed a decline of approximately 40% in deliveries and births, with the steepest reduction in the first year of the war.

The COVID-19 pandemic was associated with relatively modest and short-term changes. In contrast, the wartime period was associated with more sustained increases in maternal morbidity, including higher rates of diabetes during pregnancy, hypertensive disorders of pregnancy, and postpartum haemorrhage. Preterm birth, very low, and extremely low birthweight, and operative deliveries also increased, especially during wartime. A transient rise in uterine rupture was observed in the first year of the war. Pregnancy-related and perinatal mortality, particular stillbirths, increased during the second year of the pandemic but not during wartime. Early neonatal mortality did not change throughout.

Overall, although most relative changes were small, their cumulative impact may be important at the population level. The findings suggest that essential maternal and perinatal services were largely maintained during the war period, possibly reflecting preservation of pre-existing system organisation supported by coordinated national efforts and international aid.

**Implications of all the available evidence**

Our findings suggest that overlapping large-scale crises can place considerable strain on maternal and perinatal health systems. The resulting increase in morbidity may be substantial at the population level and may not always be fully reflected in mortality indicators alone. As conflicts and pandemic-related disruptions continue to affect Europe and other regions in the world, strengthened surveillance of maternal and perinatal outcomes may help to better identify and address excess morbidity and mortality among mothers and newborn infants. To support the development of interventions to improve maternal and perinatal outcomes in conflict settings, further research using individual-level data is needed to clarify better causal relationships between armed conflict and associated health effects. Enhancing health system resilience—including workforce capacity, referral pathways, and supply chains—may help reduce the impact of prolonged and overlapping crises on maternal and perinatal health. Healthcare systems in conflict-affected settings also require sustained attention to antenatal care, emergency obstetric services, and neonatal support, which are frequently and inequitably distributed.

## Introduction

Emergencies and humanitarian crises undermine the stability of health systems and their capacity to deliver high-quality care. There is growing evidence that maternal and perinatal outcomes deteriorate in settings of crisis and armed conflict.^1^^,^^2^

In such a context, a range of challenges intersect: rising demand for health services; disruption of routine health-system operations under unpredictable and unsafe conditions for both providers and users; damage to or destruction of infrastructure; deliberate targeting of health facilities, personnel, and ambulances; loss of trained staff; and interruptions to power and other supply chains. Collectively, these factors limit access to maternal and perinatal health services and undermine their quality, increasing the risk of adverse outcomes for mothers and newborns.^1^

Additionally, negative effects on maternal and perinatal outcomes in crises can be related to exposure of pregnant women to stress, a well-established risk factor for preterm birth,^3^ diabetes,^4^ preeclampsia,^5^ and low birthweight.^6^

In recent years, Ukraine has experienced compounding shocks from the COVID-19 pandemic and full-scale war, with potential consequences for maternal and perinatal health.

Evidence from the COVID-19 pandemic revealed a significant effect on maternal and perinatal outcomes, with global worsening of maternal and foetal health indicators, including increases in maternal mortality and stillbirths.^7^ These adverse outcomes were likely driven by health-system constraints—particularly in low-middle income countries—as well as by risks associated with SARS-CoV-2 infection.^7^

Exposure of pregnant women to conflict-related stress adversely affects maternal and neonatal health. Systematic review has reported an increased incidence of low birthweight during wartime, with more limited evidence for increases in miscarriage, stillbirth, and prematurity.^8^ Conflict-exposed populations have shown higher rates of premature rupture of membranes, preterm premature rupture of membranes, gestational diabetes, and postpartum haemorrhage.^9^ Prolonged armed conflict has also been associated with increased rates of pre-eclampsia and eclampsia.^10^ Reduced access to maternal and perinatal care during conflict has been consistently linked to higher maternal and perinatal mortality.^2^^,^^8^

The conflict in Ukraine intersected with the COVID-19 pandemic, creating concurrent humanitarian crises and placing additional strain on regional infrastructure.^11^ This dual systemic burden may have contributed to shifts in healthcare delivery and variations in maternal and perinatal health indicators.

Evidence on maternal and perinatal outcomes in the context of concurrent pandemic and large-scale armed conflict—particularly in countries with well-developed perinatal care systems—remains scarce. Given the limited national-level data on the effects of overlapping crises on maternal and perinatal health in European conflict settings, we aimed to address this gap by systematically quantifying maternal and perinatal health indicators during the concurrent COVID-19 pandemic and the war in Ukraine. This study aimed to answer research questions about how deliveries, births and perinatal indicators changed during the COVID-19 pandemic and the consecutive war in Ukraine.

## Methods

### Study design and settings

We conducted a descriptive nationwide, population-based ecological study in Ukraine, 2019–2024.

Since independence in 1991, Ukraine has provided near-universal access to skilled perinatal and obstetric care despite resource limitations. Almost all births occur in a hospital and are attended by a midwife and an obstetrician–gynaecologist.^12^ Regionalisation of perinatal services since 2003 has improved quality by concentrating expertise in tertiary centres. Healthcare reforms have been initiated since 2017 to modernise the previous Soviet healthcare system. Maternal and child health policies follow WHO principles and the Safe Motherhood initiative.^13^ National protocols are evidence-based. Since 2014, due to the occupation of Crimea and parts of the Donetsk and Luhansk regions, the system lost perinatal institutions there.

**Fig. 1:**
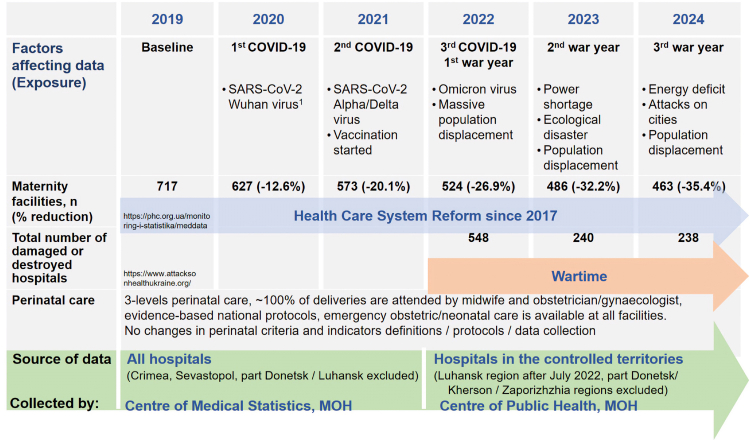
**Contextual changes during the study period, Ukraine 2019–2024.** Per cent (%) reduction - reduction compared to 2019. MOH = Ministry of Health. Four waves of the COVID-19 pandemic in Ukraine.^16^

The study was conducted during a period of marked contextual changes (Fig. 1). Despite the implementation of timely measures, the healthcare system faced structural capacity limitations during peak COVID-19 surges.^14^ Limited vaccination coverage and ineffective lockdown contributed to sustained transmission and increased mortality,^14^ with successive waves varying in transmissibility and severity. On February 24, 2022, when Russia launched its invasion, only about 36% of the population was vaccinated, and daily case numbers were at a peak.^11^ As the war escalated, the perceived threat of COVID-19 diminished relative to immediate safety concerns.^14^ The hostilities have compromised regional infrastructure, disrupting obstetric and neonatal services, causing mass population displacement, and severely depleting the healthcare workforce. Intermittent power outages and shortages of essential medical supplies^15^ further compounded theses systemic challenges. Further occupation, and the 2023 destruction of the Kakhovka dam, and ongoing attacks on energy infrastructure have further worsened living conditions and access to care. Healthcare reform has also led to the consolidation of services and the closure of smaller maternity facilities. In 2022, national data functions were transferred from the Centre of Medical Statistics, Ministry of Health (MOH), to the Centre of Public Health, MOH.

### Data source and study population

**Fig. 2:**
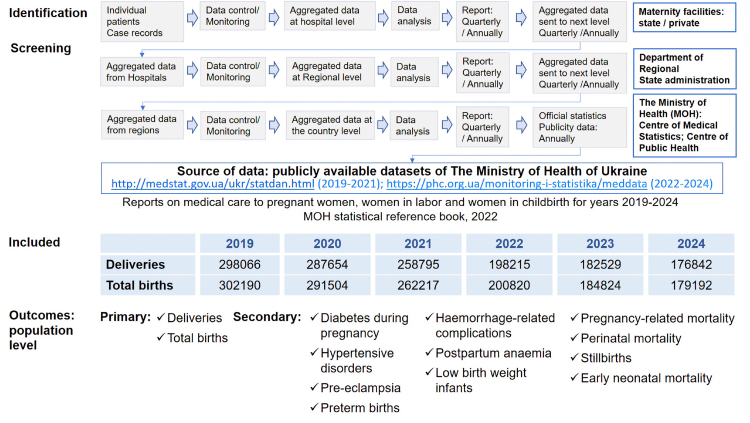
**Study flowchart.** Data presented cover the Ministry of Health (MOH), other ministries, and private institutions, except in 2021, when only the MOH data were available.

This study used routinely collected, aggregated, annual national data (Fig. 2). Data were derived from quarterly and annual reports submitted to the MOH by departments of regional state administrations, based on hospital reports.

In Ukrainian hospitals, data collection is a multilevel process. Physicians enter primary patient information (diagnoses, services, and procedures) into the electronic health system. Hospital statistics departments or designated statisticians verify, aggregate, and report data quarterly and annually. Maternity facility reports are based on individual case records and include aggregated data without patient identifiers or individual characteristics.^12^ Data undergo routine monitoring and quality control at hospital, regional, and national levels, supported by digital and remote monitoring systems.

Data from territories occupied since 2014 have been unavailable. Reporting ceased from Luhansk in summer 2022 and subsequently from occupied parts of Donetsk, Kherson, and Zaporizhzhia regions. All Government-controlled regions reported consistently throughout the study period. Official national statistics were published annually without missing data when possible.

Annual aggregated data on deliveries and births in Ukraine (2019–2024) were retrieved from publicly available MOH datasets (Form No. 21), covering the MOH, other ministries, and private maternity facilities (totalling 1,402,101 deliveries; 1,420,747 births). Data for 2021, collected in early 2022, were available only for MOH institutions, which accounted for approximately 98% of the expected records (according to estimates from 2020 to 2022). Maternal and pregnancy-related mortality data for 2019–2021 were obtained from the MOH statistical reference book.^17^ After excluding accidental deaths, maternal mortality accounted for approximately 88%, 90%, and 96% of all pregnancy-related deaths in 2019–2021. As only pregnancy-related mortality was reported in 2022–2024, pregnancy-related mortality data were used for all years. Perinatal mortality indices were based on maternity hospital data. Predefined maternal and perinatal indicators were extracted for each study year.

The number of deliveries and total births were the primary outcomes of the study.

Extracted variables included: characteristics of the study population (parity, multiple gestation deliveries, and deliveries outside medical facilities); indicators of healthcare utilisation (antenatal care [ANC] attendance) and mode of delivery (caesarean or instrumental).

Secondary maternal outcomes included: pregnancy and delivery complications: diabetes during pregnancy (including both pregestational and gestational); hypertensive disorders of pregnancy (including pre-existing hypertension with superimposed pre-eclampsia, gestational hypertension, pre-eclampsia, and eclampsia), and severe pre-eclampsia (including severe pre-eclampsia and eclampsia); haemorrhage-related complications: placental abruption, placenta praevia, uterine rupture, postpartum haemorrhage (PPH, defined as estimated blood loss ≥500 mL), severe PPH (estimated blood loss ≥1000 mL), hysterectomy for PPH, third-fourth degree perineal tears, postpartum anaemia; pregnancy-related mortality.

Secondary perinatal outcomes included: preterm births (<37 + 0 weeks' gestation); low birth weights (LBW <2500 g), very low birth weights (VLBW <1500 g), extremely low birth weights (ELBW <1000 g); high birth weights (HBW ≥3500 g); perinatal mortality.

Definitions of perinatal criteria and key indicators, as well as ICD-10 codes for maternal complications, are provided in the Supplementary Appendix (p. 3).

### Statistical analysis

Statistical analyses used descriptive and inferential methods. Percentages, rates per 1000 births, and ratios per 100,000 livebirths were calculated with corresponding 95% confidence intervals (CIs). CIs were estimated using the Wilson score method without continuity correction.^18^

The analytical framework combined longitudinal and comparative approaches. To identify patterns of outcomes over time, temporal trends were assessed using the Mann–Kendall test; when a significant trend was detected, the magnitude of change was estimated using Sen's slope. Because only annual aggregated data were available for six consecutive years and contextual conditions varied substantially across years (Fig. 1), pairwise comparisons were conducted to understand changes under these conditions better and to evaluate differences in indices between specific years, thereby reducing bias. Data for each year were compared with the baseline year (pre-pandemic, 2019) and with the preceding year. Data for the third year of war (2024) were additionally compared with the pre-war year (2021) and the first year of war (2022).

Differences in categorical outcomes between years were assessed using the χ^2^ test. Associations between study year (binary exposure) and outcomes were estimated using ORs and 95% CIs derived from 2 × 2 contingency tables. In all analyses, the earlier year was treated as the reference and the later year as the comparison. Effects Sizes (ESs) were interpreted for all maternal and neonatal outcomes, based on odds ratios (ORs) with 95% CIs and corresponding p-values.^19^ ESs are presented in colours in the Supplementary Tables S3–S6 with various shades of blue for ORs <1 and red for ORs >1. Additionally, Absolute Risk Differences (ARDs) were calculated. To estimate the absolute number of additional cases associated with the exposure, the ARD was divided by 100 when expressed as a percentage, or by 1000 when expressed per 1000, and then multiplied by the relevant denominator.

As data were available for approximately 98% of cases in 2021 (the second year of the COVID-19 pandemic), we conducted sensitivity analyses on selected secondary outcomes that showed significant changes during the pandemic to assess robustness relative to the pre-pandemic baseline under a hypothetical scenario of complete ascertainment. Denominators (total deliveries and livebirths) were increased by 2% to approximate full population coverage, while numerators were held constant, reflecting a conservative assumption that would attenuate effect estimates. Statistical significance was defined as p < 0.05. Statistical analyses were conducted using the EZR graphical user interface (version 1.54), which runs on the R platform (version 4.0.3; R Foundation for Statistical Computing, Vienna, Austria). Supplementary Data manipulations were executed independently using R version 4.5.2 (2025-10-31 ucrt, R Foundation for Statistical Computing).

### Ethics approval

This ecological study was approved by the Ethics Review Committee of Odesa National Medical University (protocol 09, Nov 12, 2025), with a waiver of informed consent for the retrospective analysis of publicly available aggregated national data. The study was conducted in accordance with the RECORD statement–checklist of items, extended from the STROBE statement, that should be reported in observational studies using routinely collected health data (Supplementary Material: RECORD Checklist).

### Role of funding source

No funding has been received to undertake this research.

## Results

The number of deliveries and births declined over the study period (Table 1), with a reduction of about 40% between 2019 and 2024 and 32% during wartime; the largest decrease occurred in the first year of the war. Nulliparity decreased over time, with very small Effect Sizes (ESs) during both the COVID-19 pandemic and the war (Table 1 and Supplementary Tables S1 and S3). Antenatal care attendance remained above 99% at all timepoints but was lower than baseline, with Absolute Risk Differences (ARDs) ranging from −0.23% to −0.37% during wartime and −0.14% during the pandemic. All ESs were small or very small. No consistent patterns were observed for multiple gestation or out-of-facility deliveries.

**Table 1:** Deliveries, births and some characteristics of the population-based samples, 2019–2024.

	Pre-pandemic	COVID-19 pandemic		Wartime			Presence of trend (p)	Sen's slope (95% CI)
	2019	2020	2021	2022	2023	2024		
Number of deliveries	298,066	287,654	258,795	198,215	182,529	176,842	0.01	−27,703 (−4719.5; −10,412)
Relative % of deliveries in 2019	100.00	96.51	86.82	66.50	61.24	59.33		
Absolute risk difference compared to 2019, %		−3.49	−13.18	−33.50	−38.76	−40.67		
Total births, n	302,190	291,504	262,217	200,820	184,824	179, 192	0.01	−28,078 (−45342; −10,686)
Relative % of births in 2019	100.00	96.46	86.77	66.45	61.16	59.30		
Absolute risk difference with 2019, %		−3.54	−13.23	−33.55	−38.84	−40.70		
**Some characteristics of the population**								
Parity, nulliparous, n (%)	133,099 (44.65)	122,320 (42.52)^a^	108,501 (41.93)^a,b^	83,147 (41.95)^a^	75,037 (41.11)^a,b^	68,503 (38.74)^a–d^	0.02	−0.00902 (−0.021; −0.0029)
Multiple gestation deliveries, n (%)	4125 (1.38)	3856 (1.34)	3425 (1.32)	2604 (1.31)^a^	2315 (1.27)^a^	2350 (1.33)	0.13	
ANC attendance, n (%)	296,257 (99.39)	285,843 (99.37)	256,842 (99.25)^a,b^	196,424 (99.10)^a,b^	180,754 (99.03)^a,b^	175,111 (99.02)^a,c,d^	0.01	−0.0009 (−0.0014; −0.0002)
Deliveries out of facilities, n (%)	327 (0.11)	310 (0.11)	312 (0.12)	206 (0.10)	213 (0.12)	174 (0.10)^c^	0.45	
Total preterm births, n (%)	16,907 (5.59)	16,151 (5.54)	15,938 (6.08)^a^	11,760 (5.86)^a,b^	10,908 (5.90)^a^	11,195 (6.25)^a–d^	0.13	

Notes. Data cover the Ministry of Health (MOH), other ministries, and private providers, except in 2021, when only the MOH data were available. Denominator for all indicators, except total preterm births, is the number of deliveries; the denominator for total preterm births is the total number of births. Differences in categorical outcomes between years were assessed using the χ^2^ test. Significant difference (p < 0.05): ^a^with pre-pandemic year (2019); ^b^with the preceding year; ^c^with pre-war year (2021); ^d^with the first year of war (2022); Temporal trends were assessed using the Mann–Kendall test. In the presence of a significant trend (p < 0.05), the Sen's slope with (95% CI) is presented. ANC = antenatal care.

### Pregnancy and delivery complications

**Fig. 3:**
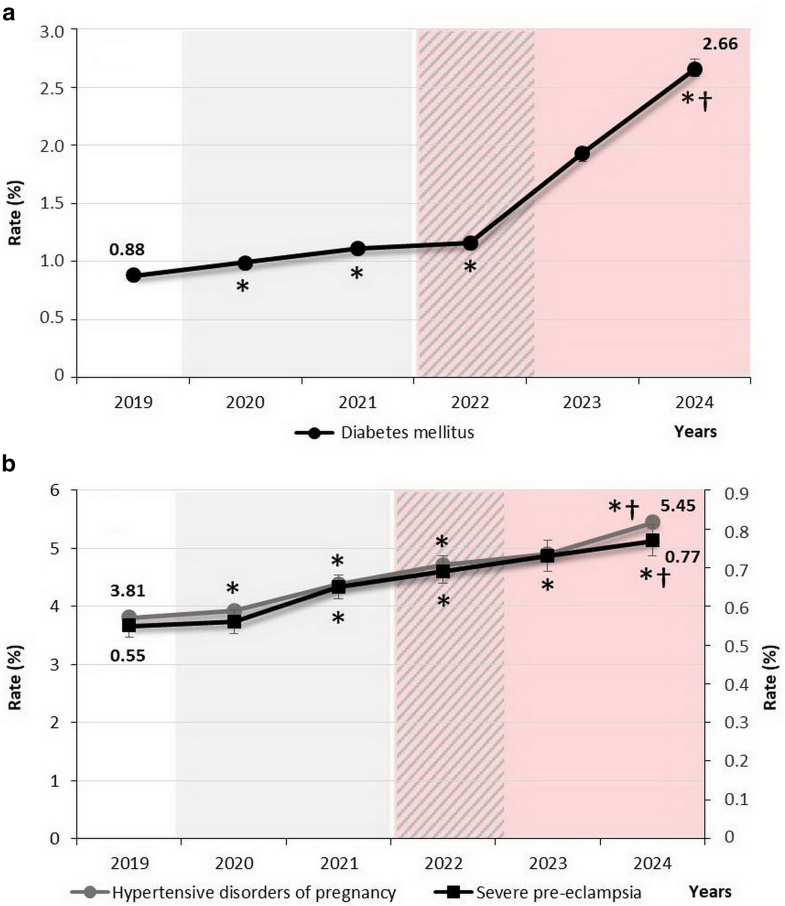
**Pregnancy and delivery complications.** Panel A shows comparative rates of diabetes mellitus during pregnancy (Mann–Kendall Test p-value = 0.009, Sen's slope with 95% CI = 0.0031 [0.0009; 0.0075]). Panel B shows comparative rates of hypertensive disorders of pregnancy (Mann–Kendall Test p-value = 0.009, Sen's slope with 95% CI = 0.0033 [0.0020; 0.0045]) and severe pre-eclampsia (Mann–Kendall Test p-value = 0.009, Sen's slope with 95% CI = 0.0005 [0.00021; 0.00066]). Shaded areas indicate the COVID-19 pandemic (2020–2021; grey) and the war period (2022–2024; pink). ∗p < 0.05 compared with 2019 (pre-pandemic); †p < 0.05 compared with 2021 (pre-war).

Diabetes during pregnancy (<1% at baseline) was more frequent at later timepoints (Fig. 3a), with ARDs of 0.11% and 0.25% during the pandemic (very small ES) and 0.28%–1.78% during the war (small to medium ESs; Supplementary Tables S1 and S3). Hypertensive disorders of pregnancy and severe pre-eclampsia were also more frequent than at baseline (Fig. 3b). ESs for hypertensive disorders were very small except in the third year of the war (small ES; ARD 1.65%). Severe pre-eclampsia showed ARDs of 0.10% and 0.22% in the second pandemic year and the third year of war, corresponding to 260 and 390 additional intensive care cases.

### Haemorrhage-related maternal complications

**Fig. 4:**
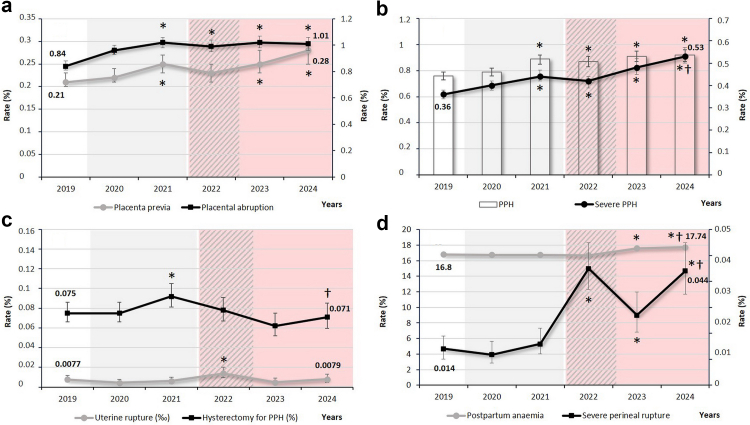
**Haemorrhage-related maternal complication.** Panel a shows comparative rates of placenta previa (Mann–Kendall Test p-value = 0.024, Sen's slope with 95% CI = 0.0001 [2.44e-05; 0.00023]) and placental abruption [Mann–Kendall Test p-value = 0.133]). Panel b shows comparative rates of postpartum haemorrhage (PPH) (Mann–Kendall Test p-value = 0.024, Sen's slope with 95% CI = 0.00032 [8.85e-05; 0.00064]) and severe PPH (Mann–Kendall Test p-value = 0.024, Sen's slope with 95% CI = 0.00033 [8.405e-05; 0.00058]). Panel c shows comparative rates of uterine rupture (Mann–Kendall Test p-value = 0.707) and hysterectomy for PPH (Mann–Kendall Test p-value = 0.452). Panel d shows comparative rates of postpartum anaemia (Mann–Kendall Test p-value = 0.452) and severe perineal rupture: third-fourth degree perineal tears (Mann–Kendall Test p-value = 0.133). Shaded areas indicate the COVID-19 pandemic (2020–2021; grey) and the war period (2022–2024; pink). ∗p < 0.05 compared with 2019 (pre-pandemic); †p < 0.05 compared with 2021 (pre-war).

Placenta praevia was more frequent at later timepoints, whereas placental abruption showed no consistent differences (Fig. 4a and b); ESs were very small (Supplementary Tables S1 and S3). Uterine rupture showed ARD of 0.006% (12 additional cases) in the first year of the war (small ES) and lower values in the second war year (medium ES; Fig. 4c). Postpartum haemorrhage (PPH) and severe PPH were more frequent than at baseline across several timepoints, with very small ESs overall and small ES for severe cases in the third year of war (ARD 0.17%; 302 additional cases).

Hysterectomy for postpartum haemorrhage showed an ARD of 0.017% in the second year of pandemic (very small ES, Fig. 4c). Third- and fourth-degree perineal tears showed ARDs of 0.031% and 0.03% in the first and third years of war (medium ESs; Fig. 4d). Postpartum anaemia showed ARDs of 0.81% and 0.96% in the last two years of war (very small ESs; 1478 and 1698 additional cases).

### Operative deliveries

**Fig. 5:**
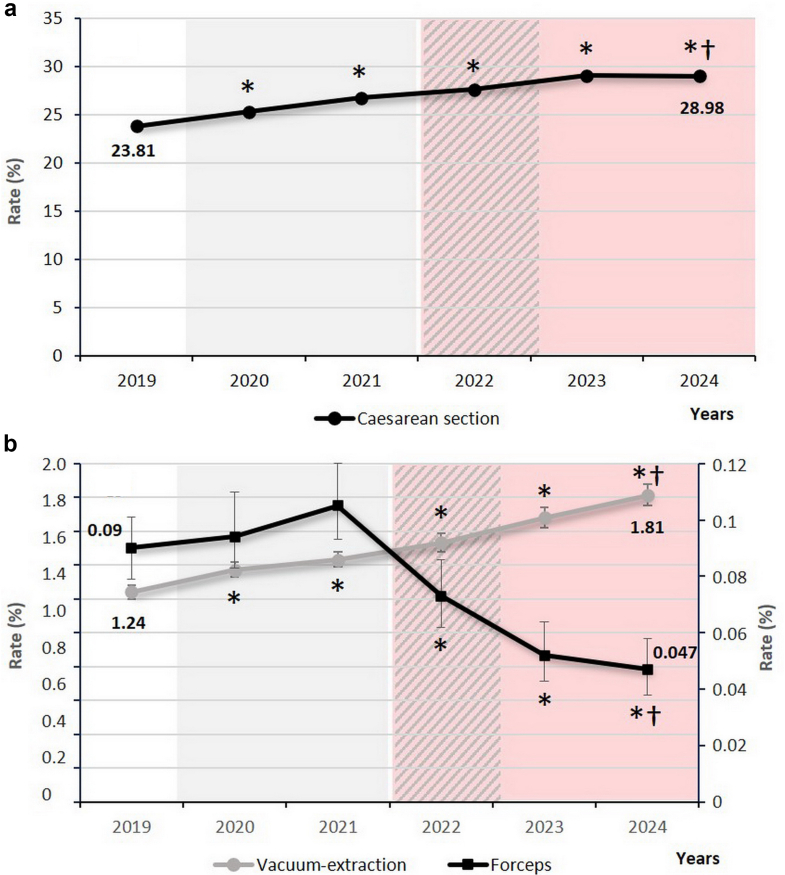
**Operative deliveries.** Panel a shows comparative rates of caesarean section (Mann–Kendall Test p-value = 0.024, Sen's slope with 95% CI = 0.012 [0.007; 0.015]). Panel b shows comparative rates of assisted vaginal delivery: vacuum extraction (Mann–Kendall Test p-value = 0.009, Sen's slope with 95% CI = 0.0011 [0.00081; 0.0014]) and forceps (Mann–Kendall Test p-value = 0.133). Shaded areas indicate the COVID-19 pandemic (2020–2021; grey) and the war period (2022–2024; pink). ∗p < 0.05 compared with 2019 (pre-pandemic); †p < 0.05 compared with 2021 (pre-war).

Approximately 30% of deliveries were by caesarean section and fewer than 2% were vacuum-assisted; both proportions were higher than baseline at multiple timepoints (Fig. 5a and b). Most differences were statistically significant with very small ESs, except for vacuum-assisted deliveries in the third year of war (small ES; ARD 0.58%). Caesarean section in the third year of war showed an ARD of 5.19%, compared with baseline, corresponding to 9178 additional procedures (very small ES vs baseline; Supplementary Tables S1 and S4). Forceps-assisted deliveries (approximately 0.1%) were less frequent during wartime (small ES).

### Perinatal outcomes: preterm birth and low birthweight

**Fig. 6:**
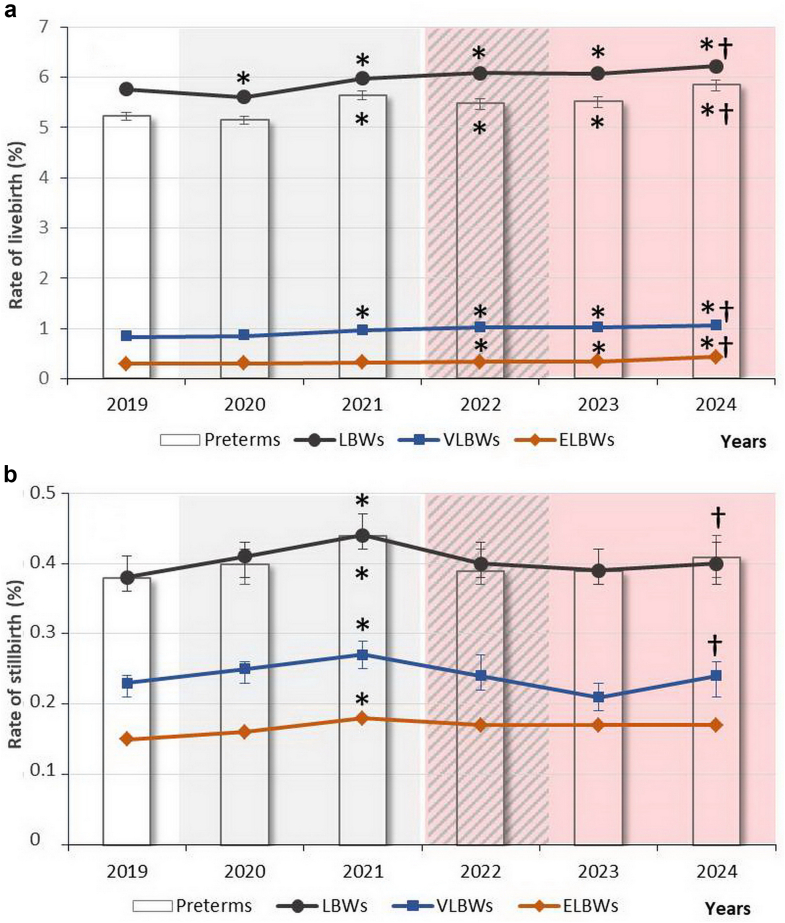
**Preterm and low-birth-weight births.** Panel a shows comparative rates of liveborn preterm (Mann–Kendall Test p-value = 0.133) and low-birth-weight (Mann–Kendall Test p-value = 0.06) infants. Panel b shows comparative rates of stillborn preterm (Mann–Kendall Test p-value = 0.707) and low-birth-weight infants (Mann–Kendall Test p-value = 1.0). Shaded areas indicate the COVID-19 pandemic (2020–2021; grey) and the war period (2022–2024; pink). ∗p < 0.05 compared with 2019 (pre-pandemic); †p < 0.05 compared with 2021 (pre-war). LBW = low-birth-weight (<2500 g), VLBW = very-low-birth-weight (<1500 g), ELBW = extremely-low-birth-weight (<1000 g).

Preterm births (5–6%) showed higher proportions from the second year of the pandemic onward, with very small ESs (Table 1 and Supplementary Tables S2 and S5). Low birthweights and very low birthweights (VLBW) showed a small increase (very small ESs), whereas extremely low birthweights showed larger relative differences (Supplementary Table S2). Among livebirths (Fig. 6a), ARDs for preterm births and VLBWs were 0.42% and 0.13% (1101 and 342 cases) in the second pandemic year and 0.62% and 0.22% (1111 and 394 cases) in the third year of the war. Extremely low birthweights showed an ARD of 0.14% (248 cases) in the third year of the war (small ES; Supplementary Table S5). Stillbirths showed no consistent differences across gestational age or birthweight categories, although they were more frequent in the second year of the pandemic (very small ES; Fig. 6b). The proportion of neonates weighing more than 3500 g remained between 38% and 40% (Supplementary Tables S2 and S6).

### Pregnancy-related and perinatal mortality

**Fig. 7:**
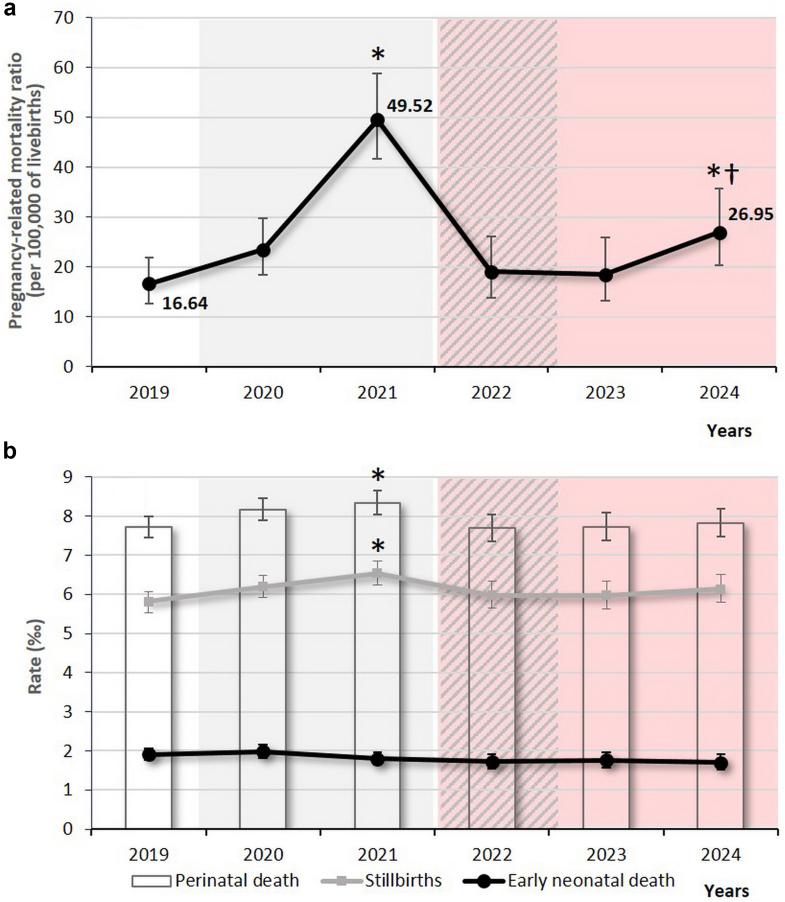
**Pregnancy-related and perinatal mortality.** Panel a shows comparative ratios of pregnancy-related mortality (per 100,000 livebirths) (Mann–Kendall Test p-value = 0.707). Panel b shows comparative rates of perinatal (Mann–Kendall Test p-value = 1.0), early neonatal (Mann–Kendall Test p-value = 0.06) deaths, and stillbirths (Mann–Kendall Test p-value = 1.0). Shaded areas indicate the COVID-19 pandemic (2020–2021; grey) and the war period (2022–2024; pink). ∗p < 0.05 compared with 2019 (pre-pandemic); †p < 0.05 compared with 2021 (pre-war). Perinatal mortality indices are based on data from maternity hospitals.

Pregnancy-related and perinatal mortality showed no consistent patterns (Supplementary Table S2). The pregnancy-related mortality showed an ARD of 32.88 per 100,000 livebirths in the second year of the pandemic (medium ES) compared with baseline; −30.48 in the first year of the war (medium ES), and 6.23 in the third year of the war compared with the first year (no significant difference; Fig. 7a; Supplementary Table S7). Perinatal mortality showed an ARD of 0.61 per 1000 in the second pandemic year (very small ES) and lower values in the first year of the war (Fig. 7b). Early neonatal mortality remained similar across timepoints, whereas stillbirths showed an ARD of 0.73 per 1000 in the second year of the pandemic (very small ES).

### Sensitivity analyses

Compared with the main analysis, sensitivity analyses under a hypothetical scenario of complete population coverage in 2021 (the second year of the COVID-19 pandemic) showed attenuated ARDs for perinatal mortality that did not retain statistical significance, with only borderline evidence of association and consistent direction of effect. ARDs for stillbirths and all other indicators were attenuated compared with the main analysis but remained statistically significant, with similar direction of effect (Supplementary Material: Sensitivity analyses).

## Discussion

Using combined longitudinal and comparative approaches, and national population-based data with Effect Sizes and Absolute Risk Differences, this study shows that the COVID-19 pandemic and the war were associated with measurable shifts in the number of deliveries, births, and maternal and perinatal indicators. The main findings include marked declines in deliveries and births between 2019 and 2024, with distinct patterns observed during the second pandemic year and the first year of the war. These findings are consistent with World Bank estimates of a decline in Ukraine's total fertility rate (1.22 in 2019; 1.15 in 2021; 0.90 in 2022—among the lowest in Europe—and 0.99 in 2024).^20^ Similar reductions in fertility have been reported globally during COVID-19.^21^ The pronounced decline observed in the first year of the war is likely multifactorial, reflecting mass displacement and large-scale international migration, family separation, limited access to birth registration data from occupied territories, and widespread insecurity and economic uncertainty. The magnitude of these reductions corroborates earlier reports from Ukraine.^13^ Changes in gestational outcomes may also be associated with maternal stress, which may have influenced both spontaneous pregnancy loss and elective terminations due to socioeconomic and security-related uncertainties; however, these mechanisms were beyond the scope of the present study. This substantial decline in live births is likely to have profound long-term consequences for Ukraine.

Our findings indicate that changes in maternal and perinatal outcomes during the COVID-19 pandemic were generally small. Despite reports of major disruptions to health services,^22^ antenatal care attendance in our study remained high (>99%). Even so, we observed potential early warning signals. In contrast to some early research,^7^ we noted an increased rate of diabetes during pregnancy, which warrants future investigation. We also saw increases in hypertensive disorders, severe pre-eclampsia, and postpartum haemorrhage. These observations align with some global reports linking SARS-CoV-2 infection to a higher risk of postpartum haemorrhage^23^ and pre-eclampsia, alongside some evidence suggesting vaccination might reduce pre-eclampsia risk.^24^ A small rise in preterm birth during the second pandemic year could potentially be related to different virus variants,^25^ though more data is needed to confirm that.

The wartime period was characterised by more consistent upward trends across several morbidity indicators, though these findings require careful interpretation. Notably, antenatal care attendance was lower during the war than in the pandemic, yet it remained above 99%, indicating sustained access to routine services, unlike other reports.^26^ While our observations regarding increases in diabetes,^9^ hypertensive disorders, severe pre-eclampsia,^10^ postpartum haemorrhage,^9^ and extremely low birthweights^8^ align with some prior conflict studies, we must consider that these findings could be partially linked to conflict-related stress. While the statistical Effect Sizes were generally small, the absolute increases in severe cases—including those requiring intensive care—appear notable. Our recorded proportion of maternal complications was substantially lower than what is typically published, which might suggest diagnostic bias or care quality issues. We found no changes in diagnostic criteria or data collection that would explain these shifts. Still, we cannot completely rule out that increased awareness among pregnant women and providers during wartime could have influenced reporting. Accordingly, our findings of increased unfavourable outcomes should be interpreted with caution and require further investigation.

Haemorrhage-related complications may indicate underlying issues in emergency care. For instance, the temporary rise in uterine rupture during the first year of the war—which later declined—occurred alongside disruption and subsequent stabilisation of referral pathways,^27^ suggesting a possible connection. A uterine scar from a previous CS, which in Ukraine accounts for more than 20% of deliveries, along with increased multiparity recoded in our study, is a well-recognised risk factor for uterine rupture in subsequent pregnancies and often requires urgent laparotomy—challenging in conflict-affected settings.^28^ Additionally, increases in severe postpartum haemorrhage, major perineal trauma, and postpartum anaemia occurred alongside reported constraints in intrapartum care, staffing, and essential supplies, including blood products.^15^ However, whether these shortages directly caused the rise in complications remains unclear. The decline in surgical PPH management during the war coincided with increased availability of non-surgical tools, including misoprostol and intra-uterine balloon tamponade devices (e.g., Ellavi systems), from humanitarian donors,^28^ but more data is needed to establish any direct link.

Operative delivery rates (CS and vacuum) increased slightly during the pandemic, whereas the wartime period was associated with higher rates of CS and vacuum-assisted delivery, alongside declining forceps use. These changes occurred amid constrained clinical environments and varying availability of personnel skilled in instrumental vaginal delivery.^29^ Future research is needed to explore if staff shortages directly influenced these changes.

Perinatal outcomes during wartime showed more unfavourable trends than during the pandemic. Increases in preterm births, very low birthweight and extremely low birthweight infants were noted, aligning with findings from other conflict settings, where maternal stress has been suggested as a contributing factor to higher neonatal risk.^30^ However, we must interpret these findings cautiously, as we cannot isolate maternal stress from other factors like nutrition or disrupted care.

Mortality patterns varied between the two periods. The pandemic period saw a temporary spike in pregnancy-related mortality and a slight rise in perinatal mortality, especially stillbirths, coinciding with changes in viral variants and overall population mortality.^14^ The attenuation of the absolute risk difference (ARD) for perinatal mortality observed in sensitivity analyses likely reflects the conservative assumption of an increased denominator with an unchanged number of events, which would be expected to dilute effect estimates. While results suggest stability despite possible underreporting, missing data may have affected the findings' strength. Significant ARDs were noted for pregnancy-related mortality and stillbirth, consistent with international meta-analyses that reported higher rates of unfavourable outcomes during COVID-19^31^; however, variations in local reporting necessitate caution in comparisons.

During the wartime period, pregnancy-related mortality did not show a sustained increase, despite worsening morbidity indicators. Stillbirths and early neonatal mortality also did not show lasting increases. While this might suggest that essential maternal and newborn services continued to function despite disruptions, we must consider alternative explanations. Similar patterns in other conflict zones have been linked to the prioritisation of emergency care or changes in service delivery. This aligns with the concept of health system resilience, where emergency interventions are preserved even as preventive and routine care drops.^22^ However, we cannot rule out that the apparent stability in mortality is a reflection of data gaps, as families fleeing conflict areas may have experienced worse outcomes that were not captured in our records.

The breakdown and disruption of healthcare infrastructure during conflict are well-documented contributors to the deterioration of maternal and perinatal health.^1^ Targeted attacks on critical infrastructure—especially energy grids providing heating during winter months—have compromised clinical environments. The resulting hypothermia is of particular concern for pregnant women, especially those undergoing emergency surgery, and for neonates. These observations underscore the importance of sustained support for maternal and neonatal health services in wartime settings.

A major strength of this study is the use of a complete national dataset on all deliveries and births over a six-year period, which allowed an assessment of temporal changes and their association with major system shocks, namely the COVID-19 pandemic and the war. The large sample size provided sufficient statistical power to detect small but clinically meaningful absolute changes in maternal and perinatal outcomes. Standardised data collection and the inclusion of multiple maternal and neonatal indicators reinforced internal validity and allowed a comprehensive assessment of health system performance over time.

However, several limitations must be considered. The completeness of the data during the war period cannot be fully verified. The observational design precludes causal inferences; residual confounding cannot be excluded, and the overlapping effects of pandemic and war cannot be fully disentangled.

The use of aggregated data introduces the risk of ecological fallacy, as population-level associations may not reflect individual-level relationships. The lack of detailed individual-level data—such as socioeconomic status, comorbidities, or access to care, including COVID-19 vaccination—limits the assessment of underlying mechanisms. Studies using individual-level data may have helped to address this limitation by better capturing the nature of these crises.

Secondary endpoint analyses were not adjusted for multiplicity, increasing the risk of type I error. Therefore, the findings should be considered exploratory, and statistically significant results—especially those with small absolute differences—should be interpreted with appropriate caution. Changes in reporting practices, diagnostic intensity, or case ascertainment, particularly during periods of health system strain, may have introduced diagnostic and reporting bias. Potential under-reporting or incomplete data may have particularly affected estimates for outcomes that require detailed clinical documentation. Rigorous quality control would be essential to minimise diagnostic bias, particularly for rarely reported complications (e.g. PPH). Although there are indications that the rise in some outcomes may extend beyond reporting effects, this remains an unproven assumption and should be framed cautiously. Population displacement and regional disruptions to services during the study period may have affected both denominators and the population risk profile. To mitigate reporting and regional-disruption bias, a population-based approach was used, incorporating a broad range of indicators regardless of the direction of the findings. At the same time, substantial migration from Ukraine may also have influenced both denominators and risk profile at the national level. Finally, generalisation of findings to other settings may be limited, given differences in health system context and their potential abilities to respond to crises, especially those involving armed conflict.

The war has placed substantial pressure on Ukraine's healthcare system, prompting broad international collaboration to support humanitarian and healthcare responses. Despite profound challenges, maternal and perinatal services appear to have remained functional across many settings,^1^ likely reflecting preservation of pre-existing system organisation supported by coordinated national efforts and international assistance. International agencies—including UNFPA, WHO, UNICEF, Maternal and Childhealth Advocacy International (MCAI) and others—have provided humanitarian, financial, and technical support to maternal and perinatal health services during the crisis. Continued attention to antenatal care, emergency obstetric services, and neonatal care remains important in conflict-affected settings.

### Conclusions

Taken together, these findings indicate that the COVID-19 pandemic was mainly associated with short-term changes in maternal and perinatal health indicators. In contrast, the war was associated with more sustained disruptions in population dynamics and with greater changes in maternal and perinatal outcomes. Although the relative changes observed during both crises were generally small, they corresponded to appreciable absolute increases in adverse events at the population level. These findings underscore the importance of future research into the mechanisms linking armed conflict with diabetes in pregnancy, hypertensive disorders of pregnancy, postpartum haemorrhage, and complications related to preterm birth and low birthweight. Such outcomes may merit greater attention within maternal and perinatal health research in Ukraine and other conflict-affected settings. The relative stability of pregnancy-related and perinatal mortality during wartime may indicate preservation of essential maternal and newborn services, potentially supported by international assistance. Continued support for antenatal care, emergency obstetric and neonatal services, and the health workforce could help mitigate the effects of prolonged, overlapping crises on maternal and newborn health.

## Contributors

All authors contributed to the conception of the study. IM and DD obtained data from the dataset at the Public Health Centre, Ministry of Health, Ukraine. All authors had access to the data; NK, VM, and DS accessed and verified the data that was handled by IM and DD, who pooled and harmonised all variables and took responsibility for the integrity and accuracy of the data. IM and DD independently conducted the statistical analyses. IM wrote the first draft of the manuscript. All authors participated in the interpretation of results, critically reviewed and edited the manuscript. All authors approved the final version and are responsible for the decision to submit for publication.

## Data sharing statement

Aggregated publicity data of all births from 2019 to 2024 in Ukraine were accessed via the Public Health Centre, Ministry of Health, Ukraine. Readers can access these datasets on the following web page https://phc.org.ua/monitoring-i-statistika/meddata.

## Editor note

The Lancet Group takes a neutral position with respect to territorial claims in published maps and institutional affiliations.

## Declaration of interests

All authors declare no competing interests.

## Appendix A Supplementary data

Supplementary Appendix Part 1

Supplementary Appendix Part 2

RECORD Checklist

Sensitive Analyses

Translated Abstract
