# Supplementary material for: Maternal and perinatal outcomes during successive and overlapping crises in Ukraine, 2019–2024: a nationwide population-based ecological study
Source: Lancet Reg Health Eur. 2026 Jul 14;68:101774. doi: 10.1016/j.lanepe.2026.101774 (PMC13382322; doi:10.1016/j.lanepe.2026.101774)
Supplement: Supplementary Appendix Part 1 [file mmc1.pdf]

Supplementary appendix to

Maternal and perinatal outcomes during successive and overlapping crises in Ukraine, 2019–2024: a nationwide population-based ecological study

Iryna Mogilevkina, Valeriia Marichereda, Natalia Khadginova, David Southall, Dmytro Dobryanskyy

Table of Contents

Some definitions and key perinatal indicators used in the manuscript 3

Definitions and perinatal criteria 3

Key perinatal indicators 3

Supplementary table S1. Pairwise comparison and trend analysis of some maternal health characteristics, 2019-2024. 4

Supplementary table S2. Pairwise comparison and trend analysis of some neonatal characteristics and perinatal and pregnancy-related mortality, 2019-2024. 5

Supplementary table S3. Some population and maternal health indices, %, Ukraine, 2019-2024. 6

Supplementary table S4. Operative deliveries, %, Ukraine, 2019-2024. 8

Supplementary table S5. Preterm births, low birth weights and high birth weights in Ukraine, %, 2019-2024. 9

Supplementary table S6. Perinatal (per 1000) and pregnancy-related (per 100 000 livebirths) mortality, Ukraine, 2019-2024. 11

Some definitions and key perinatal indicators used in the manuscript

Definitions and perinatal criteria

Number of births in this study were equal to the number of infants delivered.

Nullipara in this study referred to a pregnant woman who had never given birth to a child either a liveborn or stillborn.

Preterm birth was defined as birth before 37 completed weeks of gestation. Gestational age (in weeks) was estimated based on the interval between the first day of the last menstrual period and the date of birth and/or findings from antenatal ultrasound examination.

The perinatal period was defined as beginning at 22 completed weeks of gestation (154 days from the first day of the last menstrual period) and ending after 7 completed days of neonatal life (168 hours).[1]

The early neonatal period was defined as the time from live birth to 168 hours (7 days) of life.

Category "*Diabetes in pregnancy*" was related to ICD-10 code O24 covering "Diabetes mellitus in pregnancy, childbirth, and the puerperium". Despite this category distinguishing between preexisting Type 1 or Type 2 diabetes and gestational diabetes (GD), aggregated statistics is only available for the code without subdivision to subcodes and thus includes both pre-gestational and gestational diabetes. GD was diagnosed when a 75-g oral glucose tolerance test showed one or more pathological values after 24 weeks of gestation (The Ministry of Health (MOH) of Ukraine, Order No. 417).

Category "*Hypertensive disorders of pregnancy*" in our study included pre-existing hypertension with superimposed pre-eclampsia, gestational hypertension, pre-eclampsia and eclampsia (ICD-10 codes O11, O13, O14, and O15.1–2, respectively).

Category "*Severe pre-eclampsia*" included severe preeclampsia and eclampsia (ICD-10 codes O14.1 and O15.1–2).

Postpartum haemorrhage (PPH) was defined as blood loss of 500 mL or more, and severe PPH as blood loss of 1000 mL or more, assessed by weighing in accordance with MOH of Ukraine recommendations as described previously.[2]

Postpartum anaemia (ICD-10 code O99.0) included cases diagnosed during the postpartum period (haemoglobin <110 g/L).

Key perinatal indicators

Study population characteristics, health-care service utilisation, and maternal health outcomes were expressed as percentages of the total number of deliveries.

Total preterm births, low birthweight (LBW, <2500 g) infants, very low birthweight (VLBW, <1500 g) infants, extremely low birthweight (ELBW, <1000 g) infants, high birth weight (≥3500 g) infants were expressed as percentages of the total number of births (livebirths and stillbirths combined).

Preterm stillbirths and stillbirths in different categories of low birthweight infants were expressed as percentages of the total number of births (livebirths and stillbirths combined).

The stillbirth rate was defined as the number of stillbirths from 22 weeks of gestation (or birthweight ≥500 g) per 1000 total births (livebirths and stillbirths) in the same period.

Early neonatal mortality was defined as the number of liveborn infants, irrespective of gestational age, who died within the first 168 hours after birth per 1000 livebirths.

Perinatal mortality was defined as the number of stillbirths plus liveborn infants (born at ≥22 weeks of gestation or with birthweight ≥500 g) who died within 1–7 days after birth per 1000 total births.

The maternal mortality ratio was defined as the number of maternal deaths (deaths during pregnancy, childbirth, or within 42 days of the end of pregnancy) from any cause related to or aggravated by the pregnancy or its management but not from unintentional or incidental causes per 100 000 livebirths during the specified period.

Pregnancy-related mortality – the death of a woman while pregnant or within 42 days of termination of pregnancy, irrespective of the cause of death (obstetric and non-obstetric, including unintentional or incidental causes) per 100 000 livebirths during the specified period (https://www.sad.scot.nhs.uk/bereavement/maternal-death/).

Data presented cover the MOH, other ministries, and private institutions, except in 2021, when only MOH data were available. Perinatal mortality indices are based on data from maternity hospitals.

References

1. Ministry of Health of Ukraine. On the approval of the Instructions for determining the criteria for the perinatal period, live births and stillbirths, the Procedure for registering live births and stillbirths. 2006.

2. Mogilevkina I, Gurianov V, Lindmark G. Effectiveness of emergency obstetric care training at the regional level in Ukraine: a non-randomized controlled trial. BMC Pregnancy Childbirth [Internet]. 2022 Dec [cited 2026 Jan 20];22(1):145. Available from: https://bmcpregnancychildbirth.biomedcentral.com/articles/10.1186/s12884-022-04458-9.

Supplementary table S1. Pairwise comparison and trend analysis of some maternal health characteristics, 2019-2024.

	Pre-pandemic	COVID-19 pandemic		Wartime			Presence of trend (p)	Sen`s slope (95% CI)
	2019	2020	2021	2022	2023	2024		
Numbers of deliveries, n	298066	287654	258795	198215	182529	176842	0.01	-27703 (-4719.5; -10412)
**Pregnancy and delivery complications**								
Diabetes during pregnancy, n (%)	2634 (0.88)	2852 (0.99) [a]	2873 (1.11) [a,b]	2304 (1.16) [a]	3515 (1.93) [a,b]	4707 (2.66) [a,b,c,d]	0.01	0.0031 (0.0009; 0.0075)
Hypertensive disorders of pregnancy, n (%)	11332 (3.80)	11302 (3.93) [a]	11331 (4.38) [a,b]	9326 (4.71) [a,b]	8951 (4.90) [a,b]	9633 (5.45) [a,b,c,d]	0.01	0.0033 (0.0020; 0.0045)
Severe pre-eclampsia, n (%)	1630 (0.55)	1612 (0.56)	1687 (0.65) [a,b]	1371 (0.69) [a]	1325 (0.73) [a,b]	1365 (0.77) [a,c,d]	0.01	0.0005 (0.00021; 0.00066)
**Haemorrhage-related pregnancy and delivery complications**								
Placenta previa, n (%)	638 (0.21)	637 (0.22)	641 (0.25) [a,b]	457 (0.23)	461 (0.25) [a]	487 (0.28) [a,d]	0.02	0.0001 (2.44e-05; 0.00023)
Placental abruption, n (%)	2515 (0.84)	2771 (0.96) [a]	2632 (1.02) [a,b]	1962 (0.99) [a]	1866 (1.02) [a]	1782 (1.01) [a]	0.13	
Uterine rupture, n (%)	23 (0.008)	13 (0.005)	16 (0.006)	28 (0.014) [a,b]	9 (0.005) [b]	14 (0.008)	0.71	
Postpartum haemorrhage (PPH), n (%)	2260 (0.76)	2268 (0.79)	2292 (0.89) [a,b]	1715 (0.87) [a]	1658 (0.91) [a]	1622 (0.92) [a]	0.02	0.00032 (8.85e-05; 0.00064)
Severe PPH, n (%)	1070 (0.36)	1146 (0.40) [a]	1145 (0.44) [a,b]	823 (0.42) [a]	882 (0.48) [a,b]	941 (0.53) [a,b,c,d]	0.02	0.00033 (8.405e-05; 0.00058)
Hysterectomy for PPH, n (%)	222 (0.075)	214 (0.074)	237 (0.092) [a,b]	155 (0.078)	113 (0.062)	126 (0.071) [a,c]	0.45	
Third-fourth degree perineal tears, n (%)	43 (0.014)	34 (0.012)	41 (0.016)	89 (0.045) [a,b]	49 (0.027) [a,b]	78 (0.044) [a,b,c]	0.13	
Postpartum anaemia, n (%)	50006 (16.78)	48137 (16.73)	43315 (16.74)	33041 (16.67)	32101 (17.59) [a,b]	31375 (17.74) [a,c,d]	0.45	
**Operative delivery**								
Caesarean section, n (%)	70899 (23.79)	72659 (25.26) [a]	69102 (26.70) [a,b]	54671 (27.58) [a,b]	53021 (29.05) [a,b]	51248 (28.98) [a,c,d]	0.02	0.012 (0.007; 0.015)
Vacuum-assisted delivery, n (%)	3679 (1.23)	3944 (1.37) [a]	3698 (1.43) [a]	3037 (1.53) [a,b]	3064 (1.68) [a,b]	3206 (1.81) [a,b,c,d]	0.01	0.0011 (0.00081; 0.0014)
Forceps-assisted delivery, n (%)	269 (0.090)	269 (0.094)	271 (0.101)	144 (0.073) [a,b]	94 (0.052) [a,b]	83 (0.047) [a,c,d]	0.13	

Notes. Data cover the Ministry of Health (MOH), other ministries, and private institutions, except in 2021, when only the MOH data were available. The denominator for all indicators is the total number of deliveries. Differences in categorical outcomes between years were assessed using the χ^2 test. Significant difference ($p<0{\cdot}05$) with: [a]pre-pandemic year (2019); [b]the preceding year; [c]pre-war year (2021); [d]the first year of war (2022). Temporal trends were assessed using the Mann-Kendall test. In the presence of a significant trend ($p<0.05$), the Sen`s slope with (95% CI) is presented.

Supplementary table S2. Pairwise comparison and trend analysis of some neonatal characteristics and perinatal and pregnancy-related mortality, 2019-2024.

	Pre-pandemic	COVID-19 pandemic		Wartime			Presence of trend (p)	Sen`s slope (95% CI)
	2019	2020	2021	2022	2023	2024		
Total births	302190	291504	262217	200820	184824	179192	0.01	-28078 (-45342; -10686)
Livebirths	300433	289699	260502	199619	183720	178091	0.01	-27902 (-45040; -10734)
Preterm births, n (%)	16907 (5.59)	16151 (5.54)	15938 (6.08)[a]	11760 (5.86)[a,b]	10908 (5.90)[a]	11195 (6.25)[a,b,c,d]	0.13	
Livebirth preterms, n (%)	15768 (5.22)	14971 (5.14)	14787 (5.64)[a]	10975 (5.47)[a,b]	10192 (5.51)[a]	10465 (5.84)[a,b,c,d]	0.13	
Stillbirth, preterms, n (%)	1139 (0.38)	1180 (0.40)	1151 (0.44)[a]	787 (0.39)[b]	716 (0.39)	730 (0.41)	0.71	
Low birth weights, n (%)	18572 (6.15)	17549 (6.02)[a]	16833 (6.42)[a,b]	13012 (6.48)[a]	11939 (6.46)[a]	11858 (6.62)[a,c]	0.06	
Livebirths, low birth weights, n (%)	17410 (5.76)	16363 (5.61)[a]	15668 (5.98)[a,b]	12205 (6.08)[a]	11210 (6.07)[a]	11148 (6.22)[a,c]	0.06	
Stillbirths, low birth weights, n (%)	1162 (0.38)	1186 (0.41)	1165 (0.44)[a,b]	807 (0.40)[b]	729 (0.39)	710 (0.40)[b]	1.00	
Very low birth weights, n (%)	3184 (1.05)	3206 (1.10)	3228 (1.23)[a,b]	2530 (1.26)[a]	2267 (1.23)[a]	2305 (1.29)[a]	0.06	
Livebirths, very low birth weights, n (%)	2497 (0.83)	2489 (0.85)	2524 (0.96)[a,b]	2038 (1.01)[a]	1887 (1.02)[a]	1882 (1.05)[a,c]	0.01	0.00049 (0.00018; 0.0008)
Stillbirths, very low birth weights, n (%)	687 (0.23)	717 (0.25)	704 (0.27)[a]	492 (0.24)	380 (0.21)[b]	423 (0.24)[c]	0.71	
Extremely low birth weights, n (%)	1335 (0.44)	1361 (0.47)	1311 (0.50)[a]	996 (0.50)[a]	932 (0.50)[a]	1068 (0.60)[a,b,c,d]	0.02	0.00025 (2.15e-05; 0.0005)
Livebirths, extremely low birth weights, n (%)	888 (0.29)	886 (0.30)	841 (0.32)	663 (0.33)[a]	627 (0.34)[a]	762 (0.43)[a,b,c,d]	0.01	0.00013 (9.26e-05; 0.00048)
Stillbirths, extremely low birth weights, n (%)	447 (0.15)	475 (0.16)	470 (0.18)[a]	333 (0.17)	305 (0.17)	306 (0.17)	0.26	
High birth weight, n (%)	116166 (38.44)	115478 (39.61)[a]	101649 (38.77)[a,b]	76226 (37.96)[a,b]	71955 (38.93)[a,b]	70900 (39.57)[a,b,c,d]	0.71	
Perinatal mortality[e], n (‰)	2332 (7.72)	2378 (8.16)	2184 (8.33)[a]	1545 (7.69)[b]	1427 (7.72)	1404 (7.84)	1.00	
Stillbirths[e], n (‰)	1757 (5.81)	1805 (6.19)	1715 (6.54)[a]	1201 (5.98)[b]	1104 (5.97)	1101 (6.14)	1.00	
Early neonatal mortality[e], n (‰)	575 (1.91)	573 (1.98)	469 (1.80)	344 (1.72)	323 (1.76)	303 (1.70)	0.06	
Pregnancy-related mortality, n (per 100 000 livebirths)	50 (16.64)	68 (23.47)	129 (49.52)[a,b]	38 (19.04)[b]	34 (18.51)[a,c]	45 (25.27)	0.71	

Notes. Data cover the Ministry of Health (MOH), other ministries, and private institutions, except in 2021, when only the MOH data were available. The denominator for all indicators is the total births, except for early neonatal mortality and pregnancy-related mortality, where the denominator is live births. Differences in categorical outcomes between years were assessed using the χ^2 test. Significant difference ($p<0{\cdot}05$) with: [a]pre-pandemic year (2019); [b]the preceding year; [c]pre-war year (2021); [d]the first year of war (2022); [e]based on data from maternity hospitals. Temporal trends were assessed using the Mann-Kendall test. In the presence of a significant trend ($p<0.05$), the Sen`s slope with (95% CI) is presented. Low birth weights – <2500 g; very low birth weights – <1500 g; extremely low birth weights – <1000 g; high birth weight – ≥3500 g; ‰ – per 1000.
