# Supplementary material for: Maternal and perinatal outcomes during successive and overlapping crises in Ukraine, 2019–2024: a nationwide population-based ecological study
Source: Lancet Reg Health Eur. 2026 Jul 14;68:101774. doi: 10.1016/j.lanepe.2026.101774 (PMC13382322; doi:10.1016/j.lanepe.2026.101774)
Supplement: Supplementary Appendix Part 2 [file mmc2.pdf]

Supplementary table S3. Some population and maternal health indices, %, Ukraine, 2019-2024.

Time	Pre-pandemic	COVID-19 pandemic		Wartime			Effect size estimation, OR (95% CI)										
	2019	2020	2021	2022	2023	2024	OR$^{2020-2019}$	OR$^{2021-2019}$	OR$^{2021-2020}$	OR$^{2022-2019}$	OR$^{2022-2021}$	OR$^{2023-2019}$	OR$^{2023-2022}$	OR$^{2024-2019}$	OR$^{2024-2023}$	OR$^{2024-2021}$	OR$^{2024-2022}$
Numbers of deliveries, n	298066	287654	258795	198215	182529	176842											
Relative % of deliveries in 2019 (95% CI)	100.00	96.51 (96.44; 96.58)	86.82 (86.69; 86.95)	66.50 (66.29; 66.71)	61.24 (61.02; 61.46)	59.33 (59.10; 59.56)	ARD 3.49 (3.42; 3.56)	ARD 13.18 (13.05; 13.31)		ARD 33.50 (33.29; 33.71)		ARD 38.76 (38.54; 38.98)		ARD 40.67 (40.44; 40.899)			
Delivery out of facilities, included in Total deliveries (95% CI)	327 0.11 (0.10; 0.12)	310 0.11 (0.10; 0.12)	312 0.12 (0.11; 0.13)	206 0.10 (0.09; 0.11)	213 0.12 (0.11; 0.14)	174 0.10 (0.09; 0.12)	0.98 (0.84; 1.15) p=0.84	1.10 (0.94; 1.28) p=0.23	1.12 (0.96; 1.31) p=0.17	0.95 (0.79; 1.13) p=0.57	0.86 (0.72; 1.028) p=0.10	1.06 (0.89; 1.26) p=0.48	1.12 (0.93; 1.36) p=0.24	0.89 (0.75; 1.08) p=0.27	0.84 (0.69; 1.03) p=0.10	0.82 (0.68; 0.98) p=0.03*	0.95 (0.77; 1.16) p=0.61
Parity, nulliparous, n, %, (95% CI)	133099 44.65 (44.47; 44.83)	122320 42.52 (42.34; 42.70)	108501 41.93 (41.74; 42.12)	83147 41.95 (41.73; 42.17)	75037 41.11 (40.88; 41.34)	68503 38.74 (38.51; 38.97)	0.92 (0.91; 0.93) p<0.001*	0.89 (0.88; 0.90) p<0.001*	0.98 (0.97; 0.99) p<0.001*	0.89 (0.88; 0.91) p<0.001*	1.00 (0.99; 1.01) p=0.88	0.87 (0.86; 0.88) p<0.001*	0.97 (0.95; 0.98) p<0.001*	0.78 (0.77; 0.79) p<0.001*	0.91 (0.89; 0.92) p<0.001*	0.88 (0.87; 0.89) p<0.001*	0.88 (0.87; 0.89) p<0.001*
Multiple gestation delivery, n, %, (95% CI)	4125 1.38 (1.34; 1.42)	3856 1.34 (1.30; 1.38)	3425 1.32 (1.28; 1.37)	2604 1.31 (1.26; 1.36)	2315 1.27 (1.22; 1.32)	2350 1.33 (1.28; 1.38)	0.97 (0.93; 1.01) p=0.16	0.96 (0.91; 1.00) p=0.05	0.99 (0.94; 1.03) p=0.59	0.95 (0.90; 0.99) p=0.04*	0.99 (0.94; 1.05) p=0.79	0.92 (0.87; 0.96) p<0.001*	0.97 (0.91; 1.02) p=0.22	0.96 (0.912; 1.01) p=0.12	1.05 (0.99; 1.11) p=0.11	1.00 (0.95; 1.06) p=0.89	1.01 (0.96; 1.07) p=0.69
Antenatal care attendance, n, %, (95% CI)	296257 99.39 (99.36; 99.42)	285843 99.37 (99.34; 99.40)	256842 99.25 (99.22; 99.28)	196424 99.10 (99.06; 99.14)	180754 99.03 (98.98; 99.07)	175111 99.02 (98.97; 99.06)	0.96 (0.90; 1.03) p=0.28	0.80 (0.75; 0.86) p<0.001*	0.83 (0.78; 0.89) p<0.001*	0.67 (0.63; 0.72) p<0.001*	0.83 (0.78; 0.89) p<0.001*	0.62 (0.58; 0.66) p<0.001*	0.93 (0.87; 0.99) p=0.03*	0.62 (0.58; 0.66) p<0.001*	0.99 (0.93; 1.06) p=0.86	0.77 (0.72; 0.82) p<0.001*	0.92 (0.86; 0.97) p=0.02*
Diabetes during pregnancy, n, %, (95% CI)	2634 0.88 (0.85; 0.91)	2852 0.99 (0.95; 1.03)	2873 1.11 (1.07; 1.15)	2304 1.16 (1.11; 1.21)	3515 1.93 (1.87; 1.99)	4707 2.66 (2.59; 2.74)	1.12 (1.07; 1.19) p<0.001*	1.26 (1.19; 1.33) p<0.001*	1.12 (1.06; 1.18) p<0.001*	1.32 (1.25; 1.39) p<0.001*	1.05 (0.99; 1.11) p=0.10	2.20 (2.09; 2.32) p<0.001*	1.67 (1.58; 1.76) p<0.001*	3.07 (2.92; 3.22) p>0.001*	1.39 (1.33; 1.46) p<0.001*	2.44 (2.32; 2.55) p<0.001*	2.33 (2.21; 2.45) p<0.001
Hypertensive disorders of pregnancy, n, %, (95% CI)	11332 3.80 (3.73; 3.87)	11302 3.93 (3.86; 4.00)	11331 4.38 (4.30; 4.46)	9326 4.71 (4.62; 4.80)	8951 4.90 (4.80; 5.00)	9633 5.45 (5.35; 5.56)	1.04 (1.01; 1.06) p=0.01*	1.16 (1.13; 1.19) p<0.001*	1.12 (1.09; 1.15) p<0.001*	1.25 (1.22; 1.29) p<0.001*	1.08 (1.05; 1.11) p<0.001*	1.31 (1.27; 1.34) p<0.001*	1.04 (1.01; 1.08) p=0.004*	1.46 (1.42; 1.49) p<0.001*	1.12 (1.09; 1.15) p<0.001*	1.26 (1.22; 1.29) p<0.001*	1.17 (1.13; 1.20) p<0.001*
Severe pre-eclampsia, n, %, (95% CI)	1630 0.55 (0.52; 0.58)	1612 0.56 (0.53; 0.59)	1687 0.65 (0.62; 0.68)	1371 0.69 (0.65; 0.73)	1325 0.73 (0.69; 0.77)	1365 0.77 (0.73; 0.81)	1.03 (0.96; 1.09) p=0.49	1.19 (1.12; 1.28) p<0.001*	1.16 (1.09; 1.25) p<0.001*	1.27 (1.18; 1.36) p<0.001*	1.06 (0.99; 1.14) p=0.11	1.38 (1.28; 1.48) p<0.001*	1.09 (1.01; 1.17) p=0.03*	1.42 (1.32; 1.52) p<0.001*	1.03 (0.95; 1.11) p=0.48	1.19 (1.10; 1.27) p<0.001*	1.12 (1.04; 1.20 p=0.004*
Placenta previa, n, %, (95% CI)	638 0.21 (0.19; 0.23)	637 0.22 (0.20; 0.24)	641 0.25 (0.23; 0.27)	457 0.23 (0.21; 0.25)	461 0.25 (0.23; 0.27)	487 0.28 (0.26; 0.31)	1.04 (0.93; 1.16) p=0.56	1.16 (1.04; 1.29) p=0.01*	1.12 (1.00; 1.25) p=0.048*	1.08 (0.96; 1.22) p=0.24	0.93 (0.83; 1.05) p=0.25	1.18 (1.05; 1.33) p=0.01*	1.10 (0.96; 1.25) p=0.18	1.29 (1.14; 1.45) p<0.001*	1.091 (0.96; 1.24) p=0.18	1.11 (0.99; 1.25) p=0.08	1.20 (1.05; 1.36) p=0.01*

Time	Pre-pandemic	COVID-19 pandemic		Wartime			Effect size estimation, OR (95% CI)										
	2019	2020	2021	2022	2023	2024	OR$^{2020-2019}$	OR$^{2021-2019}$	OR$^{2021-2020}$	OR$^{2022-2019}$	OR$^{2022-2021}$	OR$^{2023-2019}$	OR$^{2023-2022}$	OR$^{2024-2019}$	OR$^{2024-2023}$	OR$^{2024-2021}$	OR$^{2024-2022}$
Placental abruption, n, %, (95% CI)	2515<br>0.84<br>(0.81;<br>0.87)	2771<br>0.96<br>(0.93;<br>0.99)	2632<br>1.02<br>(0.98;<br>1.06)	1962<br>0.99<br>(0.95;<br>1.04)	1866<br>1.02<br>(0.97;<br>1.07)	1782<br>1.01<br>(0.96;<br>1.06)	1.14<br>(1.08;<br>1.21)<br>p<0.001*	1.21<br>(1.14;<br>1.28)<br>p<0.001*	1.06<br>(1.00;<br>1.12)<br>p=0.046*	1.18<br>(1.11;<br>1.25)<br>p<0.001*	0.97<br>(0.92;<br>1.03)<br>p=0.37	1.21<br>(1.14;<br>1.29)<br>p<0.001*	1.03<br>(0.97;<br>1.10)<br>p=0.32	1.19<br>(1.13;<br>1.27)<br>p<0.001*	0.99<br>(0.92;<br>1.05)<br>p=0.67	0.99<br>(0.93;<br>1.05)<br>p=0.77	1.02<br>(0.96;<br>1.09)<br>p=0.59
Uterine rupture, n, %, (95% CI)	23<br>0.0077<br>(0.0051;<br>0.012)	13<br>0.0045<br>(0.0026;<br>0.0077)	16<br>0.0062<br>(0.0038;<br>0.01)	28<br>0.0141<br>(0.0098;<br>0.20)	9<br>0.0049<br>(0.0026;<br>0.0093)	14<br>0.0079<br>(0.0047;<br>0.013)	0.59<br>(0.30;<br>1.16)<br>p=0.16	0.80<br>(0.42;<br>1.52)<br>p=0.60	1.37<br>(0.66;<br>2.84)<br>p=0.51	1.83<br>(1.06;<br>3.18)<br>p=0.04*	2.29<br>(1.24;<br>4.22)<br>p=0.01*	0.64<br>(0.30;<br>1.38)<br>p=0.30	0.35<br>(0.17;<br>0.74)<br>p=0.01*	1.03<br>(0.53;<br>1.99)<br>p=0.92	1.61<br>(0.70;<br>3.71)<br>p=0.36	1.28<br>(0.63;<br>2.62)<br>p=0.62	0.56<br>(0.30;<br>1.06)<br>p=0.10
Postpartum haemorrhage, n, %, (95% CI)	2260<br>0.76<br>(0.73;<br>0.79)	2268<br>0.79<br>(0.76;<br>0.82)	2292<br>0.89<br>(0.85;<br>0.93)	1715<br>0.87<br>(0.83;<br>0.91)	1658<br>0.91<br>(0.87;<br>0.95)	1622<br>0.92<br>(0.88;<br>0.97)	1.04<br>(0.98;<br>1.10)<br>p=0.19	1.17<br>(1.10;<br>1.24)<br>p<0.001*	1.12<br>(1.06;<br>1.19)<br>p<0.001*	1.14<br>(1.07;<br>1.22)<br>p<0.001*	0.98<br>(0.92;<br>1.04)<br>p=0.47	1.20<br>(1.13;<br>1.28)<br>p<0.001*	1.05<br>(0.98;<br>1.12)<br>p=0.17	1.21<br>(1.14;<br>1.29)<br>p<0.001*	1.01<br>(0.94;<br>1.08)<br>p=0.79	1.04<br>(0.97;<br>1.10)<br>p=0.30	1.06<br>(0.99;<br>1.14)<br>p=0.10
Severe postpartum haemorrhage, n, %, (95% CI)	1070<br>0.36<br>(0.34;<br>0.38)	1146<br>0.39<br>(0.38;<br>0.42)	1145<br>0.44<br>(0.42;<br>0.47)	823<br>0.42<br>(0.39;<br>0.44)	882<br>0.48<br>(0.45;<br>0.52)	941<br>0.53<br>(0.49;<br>0.57)	1.11<br>(1.02;<br>1.21)<br>p=0.02*	1.23<br>(1.14;<br>1.34)<br>p<0.001*	1.11<br>(1.03;<br>1.21)<br>p=0.01*	1.16<br>(1.06;<br>1.27)<br>p=0.002*	0.94<br>(0.86;<br>1.03)<br>p=0.17	1.35<br>(1.23;<br>1.47)<br>p<0.001*	1.17<br>(1.06;<br>1.28)<br>p=0.002*	1.49<br>(1.36;<br>1.62)<br>p<0.001*	1.10<br>(1.01;<br>1.21)<br>p=0.04*	1.20<br>(1.10;<br>1.31)<br>p<0.001*	1.28<br>(1.17;<br>1.41)<br>p<0.001*
Hysterectomy for postpartum haemorrhage, n, %, (95% CI)	222<br>0.075<br>(0.066;<br>0.085)	214<br>0.074<br>(0.065;<br>0.085)	237<br>0.092<br>(0.081;<br>0.104)	155<br>0.078<br>(0.067;<br>0.091)	113<br>0.062<br>(0.052;<br>0.075)	126<br>0.071<br>(0.060;<br>0.085)	0.99<br>(0.83;<br>1.03)<br>p=0.97	1.23<br>(1.02;<br>1.48)<br>p=0.03*	1.23<br>(1.02;<br>1.48)<br>p=0.03*	1.05<br>(0.86;<br>1.29)<br>p=0.68	0.85<br>(0.70;<br>1.05)<br>p=0.13	0.83<br>(0.66;<br>1.04)<br>p=0.12	0.79<br>(0.62;<br>1.01)<br>p=0.07	0.96<br>(0.77;<br>1.19)<br>p=0.73	1.15<br>(0.89;<br>1.48)<br>p=0.31	0.78<br>(0.63;<br>0.97)<br>p=0.03*	0.91<br>(0.72;<br>1.15)<br>p=0.47
Third-fourth degree perineal tears, n, %, (95% CI)	43<br>0.014<br>(0.010;<br>0.019)	34<br>0.012<br>(0.009;<br>0.017)	41<br>0.016<br>(0.012;<br>0.022)	89<br>0.045<br>(0.037;<br>0.055)	49<br>0.027<br>(0.020;<br>0.036)	78<br>0.044<br>(0.035;<br>0.055)	0.82<br>(0.52;<br>1.29)<br>p=0.45	1.10<br>(0.72;<br>1.69)<br>p=0.75	1.34<br>(0.85;<br>2.11)<br>p=0.25	3.11<br>(2.16;<br>4.48)<br>p<0.001*	2.84<br>(1.96;<br>4.10)<br>p<0.001*	1.86<br>(1.24;<br>2.80)<br>p=0.004*	0.60<br>(0.42;<br>0.85)<br>p=0.004*	3.06<br>(2.11;<br>4.44)<br>p<0.001*	1.64<br>(1.15;<br>2.35)<br>p=0.01*	2.79<br>(1.91;<br>4.07)<br>p<0.001*	0.98<br>(0.73;<br>1.33)<br>p=0.97
Postpartum anaemia, n, %, (95% CI)	50006<br>16.78<br>(16.65;<br>16.91)	48137<br>16.73<br>(16.59;<br>16.87)	43315<br>16.74<br>(16.60;<br>16.88)	33041<br>16.67<br>(16.51;<br>16.83)	32101<br>17.59<br>(17.42;<br>17.77)	31375<br>17.74<br>(17.56;<br>17.92)	1.00<br>(0.98;<br>1.01)<br>p=0.66	1.00<br>(0.98;<br>1.01)<br>p=0.71	1.00<br>(0.99;<br>1.02)<br>p=0.96	0.99<br>(0.98;<br>1.01)<br>p=0.32	1.00<br>(0.98;<br>1.01)<br>p=0.54	1.06<br>(1.04;<br>1.08)<br>p<0.001*	1.07<br>(1.05;<br>1.09)<br>p<0.001*	1.07<br>(1.05;<br>1.09)<br>p<0.001*	1.01<br>(0.99;<br>1.03)<br>p=0.22	1.074<br>(1.06;<br>1.09)<br>p<0.001*	1.08<br>(1.06;<br>1.10)<br>p<0.001*

Notes. Data cover the Ministry of Health (MOH), other ministries, and private institutions, except in 2021, when only the MOH data were available. *Significant changes (p<0.05). OR (95% CI) – odds ratio with 95% confidential interval. In all analyses, data from the earlier year were treated as the reference and data from the later year as the comparison. Denominators are the numbers of deliveries for the corresponding year. ARD – absolute risk difference (presented only for relative % of deliveries change comparing to 2019) with 95% CI.

Effect size: OR>1 OR<1 – very small; – small; – medium.

Supplementary table S4. Operative deliveries, %, Ukraine, 2019-2024.

Time	Pre-pandemic	COVID-19 pandemic		Wartime			Effect size estimation, OR (95% CI)										
	2019	2020	2021	2022	2023	2024	$OR^{2020-2019}$	$OR^{2021-2019}$	$OR^{2021-2020}$	$OR^{2022-2019}$	$OR^{2022-2021}$	$OR^{2023-2019}$	$OR^{2023-2022}$	$OR^{2024-2019}$	$OR^{2024-2023}$	$OR^{2024-2021}$	$OR^{2024-2022}$
Numbers of deliveries, n	298066	287654	258795	198215	182529	176842											
Caesarean section, n, %, (95% CI)	70899 23.79 (23.64; 23.94)	72659 25.26 (25.10; 25.42)	69102 26.70 (26.53; 26.87)	54671 27.58 (27.38; 27.78)	53021 29.05 (28.84; 29.26)	51248 28.98 (28.77; 29.19)	1.08 (1.07; 1.10) p<0.001*	1.17 (1.15; 1.18) p<0.001*	1.08 (1.07; 1.09) p<0.001*	1.22 (1.21; 1.24) p<0.001*	1.05 (1.03; 1.06) p<0.001*	1.31 (1.30; 1.33) p<0.001*	1.08 (1.06; 1.09) p<0.001*	1.31 (1.29; 1.33) p<0.001*	1.00 (0.98; 1.01) p=0.65	1.12 (1.11; 1.14) p<0.001*	1.07 (1.06; 1.09) p<0.001*
Vacuum-assisted delivery, n, %, (95% CI)	3679 1.23 (1.19; 1.27)	3944 1.37 (1.33; 1.41)	3698 1.43 (1.39; 1.48)	3037 1.53 (1.48; 1.59)	3064 1.68 (1.62; 1.74)	3206 1.81 (1.75; 1.87)	1.11 (1.06; 1.16) p<0.001*	1.16 (1.11; 1.22) p<0.001*	1.04 (1.00; 1.09) p=0.07	1.25 (1.19; 1.31) p<0.001*	1.07 (1.02; 1.13) p=0.004*	1.37 (1.30; 1.43) p<0.001*	1.10 (1.04; 1.15) p<0.001*	1.48 (1.41; 1.55 p<0.001*	1.08 (1.03; 1.14) p=0.002*	1.27 (1.21; 1.34) p<0.001*	1.19 (1.13; 1.25) p<0.001*
Forceps-assisted delivery, n, %, (95% CI)	269 0.090 (0.080; 0.101)	269 0.094 (0.083; 0.106)	271 0.105 (0.093; 0.118)	144 0.073 (0.062; 0.086)	94 0.052 (0.043; 0.064	83 0.047 (0.038; 0.058)	1.04 (0.88; 1.23) p=0.71	1.16 (0.98; 1.37) p=0.09	1.12 (0.95; 1.33) p=0.20	0.81 (0.66; 0.99) p=0.04*	0.69 (0.57; 0.85) p<0.001*	0.57 (0.45; 0.72) p<0.001*	0.71 (0.55; 0.92) p=0.01*	0.52 (0.41; 0.67) p<0.001*	0.91 (0.68; 1.22) p=0.59	0.45 (0.35; 0.57) p<0.001*	0.65 (0.49; 0.85) p=0.002*

Notes. Data cover the Ministry of Health (MOH), other ministries, and private institutions, except in 2021, when only the MOH data were available. *Significant changes (p<0.05). OR (95% CI) – odds ratio with 95% confidential interval. In all analyses, data from the earlier year were treated as the reference and data from the later year as the comparison. Denominators are the numbers of deliveries for the corresponding year.

Effect size: OR>1 OR<1 – very small; – small.

Supplementary table S5. Preterm births, low birth weights and high birth weights in Ukraine, %, 2019-2024.

Time	Pre-pandemic	COVID-19 pandemic		Wartime			Effect size estimation, OR (95% CI)										
	2019	2020	2021	2022	2023	2024	$OR^{2020-2019}$	$OR^{2021-2019}$	$OR^{2021-2020}$	$OR^{2022-2019}$	$OR^{2022-2021}$	$OR^{2023-2019}$	$OR^{2023-2022}$	$OR^{2024-2019}$	$OR^{2024-2023}$	$OR^{2024-2021}$	$OR^{2024-2022}$
Total births, n	302190	291504	262217	200820	184824	179192											
**Preterm births**																	
Total preterm births, n, %, (95% CI)	16907<br>5.59<br>(5.51;<br>5.68)	16151<br>5.54<br>(5.46;<br>5.62)	15938<br>6.08<br>(5.99;<br>6.17)	11760<br>5.86<br>(5.75;<br>5.96)	10908<br>5.90<br>(5.80;<br>6.01)	11195<br>6.25<br>(6.14;<br>6.36)	0.99<br>(0.97;<br>1.01)<br>p=0.37	1.09<br>(1.07;<br>1.12)<br>p<0.001*	1.10<br>(1.08;<br>1.13)<br>p<0.001*	1.05<br>(1.02;<br>1.08)<br>p<0.001*	0.96<br>(0.94;<br>0.99)<br>p=0.002*	1.06<br>(1.03;<br>1.09)<br>p<0.001*	1.01<br>(0.98;<br>1.04)<br>p=0.55	1.12<br>(1.10;<br>1.15)<br>p<0.001*	1.06<br>(1.03;<br>1.09)<br>p<0.001*	1.03<br>(1.00;<br>1.06)<br>p=0.02*	1.07<br>(1.04;<br>1.10)<br>p<0.001*
Preterm live births, n, %, (95% CI)	15768<br>5.22<br>(5.14;<br>5.30)	14971<br>5.14<br>(5.06;<br>5.22)	14787<br>5.64<br>(5.55;<br>5.73)	10975<br>5.47<br>(5.37;<br>5.57)	10192<br>5.51<br>(5.41;<br>5.62)	10465<br>5.84<br>(5.73;<br>5.95)	0.98<br>(0.96;<br>1.01)<br>p=0.16	1.09<br>(1.06;<br>1.11)<br>p<0.001*	1.10<br>(1.08;<br>1.13)<br><0.001*	1.05<br>(1.02;<br>1.08)<br>p<0.001*	0.97<br>(0.94;<br>0.99)<br>p=0.002*	1.06<br>(1.03;<br>1.09)<br>p<0.001*	1.01<br>(0.98;<br>1.04)<br>p=0.55	1.13<br>(1.10;<br>1.16)<br>p<0.001*	1.06<br>(1.03;<br>1.09)<br>p<0.001*	1.04<br>(1.01;<br>1.07)<br>p=0.002*	1.07<br>(1.04;<br>1.10)<br>p<0.001*
Preterm stillbirths, n, %, (95% CI)	1139<br>0.38<br>(0.36;<br>0.40)	1180<br>0.41<br>(0.38;<br>0.43)	1151<br>0.44<br>(0.42;<br>0.47)	787<br>0.39<br>(0.37;<br>0.42)	716<br>0.39<br>(0.36;<br>0.42)	730<br>0.41<br>(0.38;<br>0.44)	1.07<br>(0.99;<br>1.17)<br>p=0.09	1.17<br>(1.07;<br>1.27)<br>p<0.001*	1.09<br>(1.00;<br>1.18)<br>p=0.05	1.04<br>(0.95;<br>1.14)<br>p=0.40	0.89<br>(0.82;<br>0.98)<br>p=0.01*	1.03<br>(0.94;<br>1.13)<br>p=0.58	0.99<br>(0.89;<br>1.09)<br>p=0.84	1.08<br>(0.99;<br>1.19)<br>p=0.10	1.05<br>(0.95;<br>1.17)<br>p=0.34	0.93<br>(0.85;<br>1.02)<br>p=0.12	1.04<br>(0.94;<br>1.15)<br>p=0.46
**Low birth weight infants**																	
<2500 g total, n, %, (95% CI)	18572<br>6.15<br>(6.06;<br>6.23)	17549<br>6.02<br>(5.93;<br>6.11)	16833<br>6.42<br>(6.33;<br>6.51)	13012<br>6.48<br>(6.37;<br>6.59)	11939<br>6.46<br>(6.35;<br>6.57)	11858<br>6.62<br>(6.50;<br>6.73)	0.98<br>(0.96;<br>1.00)<br>p=0.04*	1.05<br>(1.03;<br>1.07)<br>p<0.001*	1.07<br>(1.05;<br>1.10)<br>p<0.001*	1.06<br>(1.03;<br>1.08)<br>p<0.001*	1.01<br>(0.99;<br>1.03)<br>p=0.41	1.06<br>(1.03;<br>1.08)<br>p<0.001*	1.00<br>(0.97;<br>1.02)<br>p=0.81	1.08<br>(1.06;<br>1.11)<br>p<0.001*	1.03<br>(1.00;<br>1.05)<br>p=0.06	1.03<br>(1.01;<br>1.06)<br>p=0.01*	1.02<br>(1.00;<br>1.05)<br>p=0.09
<2500 g live births, n, %, (95% CI)	17410<br>5.76<br>(5.68;<br>5.84)	16363<br>5.61<br>(5.53;<br>5.69)	15668<br>5.98<br>(5.89;<br>6.07)	12205<br>6.08<br>(5.97;<br>6.18)	11210<br>6.07<br>(5.96;<br>6.17)	11148<br>6.22<br>(6.11;<br>6.33)	0.97<br>(0.95;<br>0.99)<br>p=0.01*	1.04<br>(1.02;<br>1.06)<br>p<0.001*	1.07<br>(1.05;<br>1.09)<br>p<0.001*	1.06<br>(1.03;<br>1.08)<br>p<0.001*	1.02<br>(0.99;<br>1.04)<br>p=0.15	1.06<br>(1.03;<br>1.08)<br>p<0.001*	1.00<br>(0.97;<br>1.03)<br>p=0.89	1.09<br>(1.06;<br>1.11)<br>p<0.001*	1.03<br>(1.00;<br>1.06)<br>p=0.05	1.04<br>(1.02;<br>1.07)<br>p<0.001*	1.03<br>(1.00;<br>1.05)<br>p=0.07
<2500 g stillbirths, n, %, (95% CI)	1162<br>0.38<br>(0.36;<br>0.40)	1186<br>0.41<br>(0.39;<br>0.43)	1165<br>0.44<br>(0.42;<br>0.47)	807<br>0.40<br>(0.38;<br>0.43)	729<br>0.39<br>(0.37;<br>0.42)	710<br>0.40<br>(0.37;<br>0.43)	1.06<br>(1.00;<br>1.15)<br>p=0.18	1.16<br>(1.07;<br>1.25)<br>p<0.001*	1.09<br>(1.01;<br>1.19)<br>p=0.03*	1.05<br>(0.96;<br>1.14)<br>p=0.36	0.90<br>(0.83;<br>0.99)<br>p=0.03*	1.03<br>(0.94;<br>1.13)<br>p=0.61	0.98<br>(0.89;<br>1.09)<br>p=0.73	1.03<br>(0.94;<br>1.13)<br>p=0.54	1.01<br>(0.91;<br>1.11)<br>p=0.95	0.89<br>(0.81;<br>0.98)<br>p=0.02*	0.99<br>(0.89;<br>1.09)<br>p=0.80
**Very low birth weight infants**																	
<1500 g total, n, %, (95% CI)	3184<br>1.05<br>(1.02;<br>1.09)	3206<br>1.10<br>(1.09;<br>1.11)	3228<br>1.23<br>(1.19;<br>1.27)	2530<br>1.26<br>(1.21;<br>1.31)	2267<br>1.23<br>(1.18;<br>1.28)	2305<br>1.29<br>(1.24;<br>1.34)	1.04<br>(0.99;<br>1.10)<br>p=0.09	1.17<br>(1.11;<br>1.23)<br>p<0.001*	1.12<br>(1.07;<br>1.18)<br>p<0.001*	1.20<br>(1.14;<br>1.26)<br>p<0.001*	1.02<br>(0.97;<br>1.08)<br>p=0.39	1.17<br>(1.11;<br>1.23)<br>p<0.001*	0.97<br>(0.92;<br>1.03)<br>p=0.36	1.22<br>(1.16;<br>1.29)<br>p<0.001*	1.05<br>(0.99;<br>1.11)<br>p=0.11	1.05<br>(0.99;<br>1.10)<br>p=0.11	1.02<br>(1.00;<br>1.08)<br>p=0.48
<1500 g live births, n, %, (95% CI)	2497<br>0.83<br>(0.79;<br>0.86)	2489<br>0.85<br>(0.82;<br>0.89)	2524<br>0.96<br>(0.92;<br>1.00)	2038<br>1.01<br>(0.97;<br>1.06)	1887<br>1.02<br>(0.98;<br>1.07)	1882<br>1.05<br>(1.00;<br>1.10)	1.03<br>(0.98;<br>1.09)<br>p=0.25	1.17<br>(1.10;<br>1.23)<br>p<0.001*	1.13<br>(1.07;<br>1.19)<br>p<0.001*	1.23<br>(1.16;<br>1.31)<br>p<0.001*	1.06<br>(1.00;<br>1.12)<br>p=0.08	1.24<br>(1.17;<br>1.32)<br>p<0.001*	1.01<br>(0.95;<br>1.07)<br>p=0.86	1.27<br>(1.20;<br>1.35)<br>p<0.001*	1.03<br>(0.97;<br>1.10)<br>p=0.39	1.09<br>(1.03;<br>1.16)<br>p=0.004*	1.04<br>(0.97;<br>1.10)<br>p=0.29

Time	Pre-pandemic	COVID-19 pandemic		Wartime			Effect size estimation, OR (95% CI)										
	**2019**	**2020**	**2021**	**2022**	**2023**	**2024**	$OR^{2020-2019}$	$OR^{2021-2019}$	$OR^{2021-2020}$	$OR^{2022-2019}$	$OR^{2022-2021}$	$OR^{2023-2019}$	$OR^{2023-2022}$	$OR^{2024-2019}$	$OR^{2024-2023}$	$OR^{2024-2021}$	$OR^{2024-2022}$
<1500 g stillbirths, n, %, (95% CI)	687<br>0.23<br>(0.21; 0.25)	717<br>0.25<br>(0.23; 0.27)	704<br>0.27<br>(0.25; 0.29)	492<br>0.24<br>(0.22; 0.27)	380<br>0.21<br>(0.19; 0.23)	423<br>0.24<br>(0.21; 0.26)	1.08<br>(0.97; 1.20)<br>p=0.15	1.18<br>(1.06; 1.31)<br>p=0.002*	1.09<br>(0.98; 1.21)<br>p=0.10	1.08<br>(0.96; 1.21)<br>p=0.22	0.91<br>(0.81; 1.02)<br>p=0.13	0.90<br>(0.80; 1.03)<br>p=0.12	0.84<br>(0.73; 0.96)<br>p=0.01*	1.04<br>(0.92; 1.17)<br>p=0.56	1.15<br>(1.00; 1.32)<br>p=0.05	0.88<br>(0.78; 0.99)<br>p=0.04*	0.96<br>(0.85; 1.10)<br>p=0.60
**Extremely low birth weight infants**																	
<1000 g total, n, %, (95% CI)	1335<br>0.44<br>(0.42; 0.46)	1361<br>0.47<br>(0.44; 0.49)	1311<br>0.50<br>(0.47; 0.53)	996<br>0.50<br>(0.47; 0.53)	932<br>0.50<br>(0.47; 0.54)	1068<br>0.60<br>(0.56; 0.63)	1.06<br>(0.98; 1.14)<br>p=0.16	1.13<br>(1.05; 1.22)<br>p=0.002*	1.07<br>(0.99; 1.16)<br>p=0.08	1.12<br>(1.04; 1.22)<br>p=0.01*	0.99<br>(0.91; 1.08)<br>p=0.87	1.14<br>(1.05; 1.24)<br>p=0.002*	1.02<br>(0.93; 1.11)<br>p=0.73	1.35<br>(1.25; 1.47)<br>p<0.001*	1.18<br>(1.08; 1.29)<br>p<0.001*	1.19<br>(1.10; 1.29)<br>p<0.001*	1.20<br>(1.10; 1.31)<br>p<0.001*
<1000 g live births, n, %, (95% CI)	888<br>0.29<br>(0.27; 0.31)	886<br>0.30<br>(0.28; 0.32)	841<br>0.32<br>(0.30; 0.34)	663<br>0.33<br>(0.31; 0.36)	627<br>0.34<br>(0.31; 0.37)	762<br>0.43<br>(0.40; 0.46)	1.03<br>(0.94; 1.14)<br>p=0.49	1.09<br>(0.99; 1.20)<br>p=0.07	1.06<br>(0.96; 1.16)<br>p=0.27	1.12<br>(1.02; 1.24)<br>p=0.03*	1.03<br>(0.93; 1.14)<br>p=0.60	1.16<br>(1.04; 1.28)<br>p=0.01*	1.03<br>(0.92; 1.15)<br>p=0.45	1.45<br>(1.32; 1.60)<br>p<0.001*	1.26<br>(1.13; 1.40)<br>p<0.001*	1.33<br>(1.20; 1.46)<br>p<0.001*	1.29<br>(1.16; 1.43)<br>p<0.001*
<1000 g stillbirths, n, %, (95% CI)	447<br>0.15<br>(0.14; 0.16)	475<br>0.16<br>(0.15; 0.18)	470<br>0.18<br>(0.16; 0.197)	333<br>0.17<br>(0.15; 0.18)	305<br>0.17<br>(0.15; 0.18)	306<br>0.17<br>(0.15; 0.19)	1.10<br>(0.97; 1.25)<br>p=0.15	1.21<br>(1.07; 1.38)<br>p=0.004*	1.10<br>(0.97; 1.25)<br>p=0.15	1.12<br>(0.97; 1.29)<br>p=0.12	0.92<br>(0.80; 1.07)<br>p=0.29	1.12<br>(0.97; 1.29)<br>p=0.15	1.00<br>(0.85; 1.16)<br>p=0.98	1.16<br>(1.00; 1.34)<br>p=0.06	1.04<br>(0.88; 1.21)<br>p=0.70	0.95<br>(0.83; 1.10)<br>p=0.53	1.03<br>(0.88; 1.20)<br>p=0.74
**High birth weight infants**																	
≥3500 g total, n, %, (95% CI)	116166<br>38.44<br>(38.27; 38.61)	115478<br>39.61<br>(39.44; 39.79)	101649<br>38.77<br>(38.58; 38.95)	76226<br>37.96<br>(37.75; 38.17)	71955<br>38.93<br>(38.71; 39.15)	70900<br>39.57<br>(39.34; 39.79)	1.05<br>(1.04; 1.06)<br>p<0.001*	1.01<br>(1.00; 1.03)<br>p=0.01*	0.97<br>(0.96; 0.98)<br>p<0.001*	0.98<br>(0.97; 0.99)<br>p<0.001*	0.97<br>(0.96; 0.98)<br>p<0.001*	1.02<br>(1.01; 1.03)<br>p<0.001*	1.04<br>(1.03; 1.06)<br>p<0.001*	1.05<br>(1.04; 1.06)<br>p<0.001*	1.03<br>(1.01; 1.04)<br>p<0.001*	1.03<br>(1.02; 1.05)<br>p<0.001*	1.07<br>(1.06; 1.08)<br>p<0.001*

Notes. Data cover the Ministry of Health (MOH), other ministries, and private institutions, except in 2021, when only the MOH data were available. *Significant changes (p<0.05). OR (95% CI) – odds ratio with 95% confidential interval. In all analyses, data from the earlier year were treated as the reference and data from the later year as the comparison. Denominators are total births for the corresponding year.

Effect size: OR>1 OR<1 – very small; – small.

Supplementary table S6. Perinatal (per 1000) and pregnancy-related (per 100 000 livebirths) mortality, Ukraine, 2019-2024.

Time	Pre-pandemic	COVID-19 pandemic		Wartime			Effect size estimation, OR (95% CI)										
	2019	2020	2021	2022	2023	2024	$OR^{2020-2019}$	$OR^{2021-2019}$	$OR^{2021-2020}$	$OR^{2022-2019}$	$OR^{2022-2021}$	$OR^{2023-2019}$	$OR^{2023-2022}$	$OR^{2024-2019}$	$OR^{2024-2023}$	$OR^{2024-2021}$	$OR^{2024-2022}$
Total births, n	302190	291504	262217	200820	184824	179192											
Relative % of births in 2019 (95% CI)	100.00	96.46<br>(96.40;<br>96.53)	86.77<br>(86.65;<br>86.89)	66.45<br>(66.29;<br>66.62)	61.16<br>(60.99;<br>61.34)	59.30<br>(59.12;<br>59.47)	ARD<br>3.54<br>(3.47;<br>3.61)	ARD<br>13.23<br>(13.10;<br>13.36)		ARD<br>33.55<br>(33.34;<br>33.76)		ARD<br>38.84<br>(38.62;<br>39.062)		ARD<br>40.70<br>(40.47;<br>40.93)			
Livebirths, n	300433	289699	260502	199619	183720	178091											
Relative % of livebirths in 2019 (95% CI)	100.00	96.43<br>(96.36;<br>96.49)	86.71<br>(86.59;<br>86.83)	66.44<br>(66.27;<br>66.61)	61.15<br>(60.98;<br>61.33)	59.28<br>(59.10;<br>59.45)	ARD<br>3.57<br>(3.50;<br>3.64)	ARD<br>13.29<br>(13.16;<br>13.42)		ARD<br>33.56<br>(33.35;<br>33.77)		ARD<br>38.85<br>(38.63;<br>39.073)		ARD<br>40.72<br>(40.49;<br>40.95)			
Stillbirths[a], n, per 1000 total births (95% CI)	1757<br>5.81<br>(5.54;<br>6.08)	1805<br>6.19<br>(5.91;<br>6.48)	1715<br>6.54<br>(6.24;<br>6.86)	1201<br>5.98<br>(5.65;<br>6.33)	1104<br>5.97<br>(5.63;<br>6.33)	1101<br>6.14<br>(5.79;<br>6.51)	1.07<br>(1.00;<br>1.14)<br>p=0.06	1.13<br>(1.05;<br>1.20)<br>p<0.001*	1.06<br>(0.99;<br>1.13)<br>p=0.11	1.03<br>(0.96;<br>1.11)<br>p=0.46	0.91<br>(0.85;<br>0.98)<br>p=0.02*	1.03<br>(0.95;<br>1.11)<br>p=0.49	1.00<br>(0.92;<br>1.08)<br>p=0.99	1.06<br>(0.98;<br>1.14)<br>p=0.16	1.03<br>(0.95;<br>1.12)<br>p=0.52	0.94<br>(0.87;<br>1.01)<br>p=0.11	1.03<br>(0.95;<br>1.12)<br>p=0.53
Early neonatal mortality[a], n, per 1000 livebirths (95% CI)	575<br>1.91<br>(1.76;<br>2.07)	573<br>1.98<br>(1.83;<br>2.13)	469<br>1.80<br>(1.64;<br>1.97)	344<br>1.72<br>(1.55;<br>1.91)	323<br>1.76<br>(1.58;<br>1.96)	303<br>1.70<br>(1.52;<br>1.90)	1.03<br>(0.92;<br>1.16)<br>p=0.60	0.94<br>(0.83;<br>1.06)<br>p=0.34	0.91<br>(0.81;<br>1.03)<br>p=0.14	0.90<br>(0.79;<br>1.03)<br>p=0.13	0.96<br>(0.83;<br>1.10)<br>p=0.56	0.92<br>(0.80;<br>1.05)<br>p=0.24	1.02<br>(0.88;<br>1.19)<br>p=0.82	0.89<br>(0.77;<br>1.02)<br>p=0.10	0.97<br>(0.83;<br>1.13)<br>p=0.71	0.95<br>(0.82;<br>1.09)<br>p=0.47	0.99<br>(0.85;<br>1.15)<br>p=0.90
Perinatal mortality[a], n, per 1000 total birth, (95% CI)	2332<br>7.72<br>(7.41;<br>8.04)	2378<br>8.16<br>(7.84;<br>8.49)	2184<br>8.33<br>(7.99;<br>8.69)	1545<br>7.69<br>(7.32;<br>8.08)	1427<br>7.72<br>(7.33;<br>8.13)	1404<br>7.84<br>(7.44;<br>8.26)	1.06<br>(1.00;<br>1.12)<br>p=0.06	1.08<br>(1.02;<br>1.15)<br>p=0.01*	1.02<br>(0.96;<br>1.08)<br>p=0.49	1.00<br>(0.94;<br>1.06)<br>p=0.93	0.92<br>(0.87;<br>0.97)<br>p=0.02*	1.00<br>(0.94;<br>1.07)<br>p=0.99	1.00<br>(0.93;<br>1.08)<br>p=0.92	1.02<br>(0.95;<br>1.09)<br>p=0.66	1.02<br>(0.94;<br>1.09)<br>p=0.71	0.94<br>(0.88;<br>1.01)<br>p=0.07	1.02<br>(0.95;<br>1.10)<br>p=0.63
Pregnancy-related mortality, n, per 100000 livebirths (95% CI)	50<br>16.64<br>(12.62;<br>21.94)	68<br>23.47<br>(18.52;<br>29.75)	129<br>49.52<br>(41.68;<br>58.83)	38<br>19.04<br>(13.87;<br>26.13)	34<br>18.51<br>(13.25;<br>25.86)	45<br>25.27<br>(20.33;<br>35.73)	1.41<br>(0.98;<br>2.03)<br>p=0.07	2.98<br>(2.15;<br>4.13)<br>p<0.001*	2.11<br>(1.57;<br>2.83)<br>p<0.001*	1.14<br>(0.75;<br>1.74)<br>p=0.59	0.38<br>(0.27;<br>0.55)<br>p<0.001*	1.11<br>(0.72;<br>1.72)<br>p=0.65	0.97<br>(0.61;<br>1.54)<br>p=1.00	1.52<br>(1.02;<br>2.27)<br>p=0.04*	1.37<br>(0.88;<br>2.13)<br>p=0.18	0.51<br>(0.36;<br>0.72)<br>p<0.001*	1.33<br>(0.86;<br>2.04)<br>p=0.23

Notes. Data cover the Ministry of Health (MOH), other ministries, and private institutions, except in 2021, when only the MOH data were available. *Significant changes (p<0.05). OR (95% CI) – odds ratio with 95% confidential interval. In all analyses, data from the earlier year were treated as the reference and data from the later year as the comparison. [a]Based on the data from maternity hospitals. Denominator for all indicators is total births for the corresponding year, except Early neonatal mortality and pregnancy-related mortality, where denominator is livebirths. ARD – absolute risk difference (presented only for relative % of total births and livebirths change, comparing to 2019) with 95% CI.

Effect size: OR>1 OR<1 – very small; – small; – medium.
