# Supplementary material for: Maternal and perinatal outcomes during successive and overlapping crises in Ukraine, 2019–2024: a nationwide population-based ecological study
Source: Lancet Reg Health Eur. 2026 Jul 14;68:101774. doi: 10.1016/j.lanepe.2026.101774 (PMC13382322; doi:10.1016/j.lanepe.2026.101774)
Supplement: Sensitive Analyses [file mmc4.pdf]

Sensitivity analyses

Denominators were increased to approximate full population coverage, while numerators were held constant, representing a conservative assumption that would attenuate effect estimates.

	Pre-pandemic, 2019	Second year of the pandemic, 2021	Effect size estimation, OR (95% CI)	p-value	ARDs, Sensitivity analyses	ARDs, Main analyses
Number of deliveries, n	298066	264077[a]				
Livebirths, n	300433	265818[a]				
Total births, n	302190	267533[b]				
Preterm births, n, per 100 total births	16907 (5.59)	15938 (5.96)	1.07 (1.05; 1.09)	<0.001	0.37	0.49
Stillbirth, n per 1000 total births	1757 (5.81)	1715 (6.41)	1.10 (1.03; 1.18)	0.004	0.6	0.73
Early neonatal mortality, n, per 1000 livebirths	575 (1.91)	469 (1.76)	0.92 (0.81; 1.04)	0.167	-0.15	-0.11
Perinatal mortality, n, per 1000 total births	2332 (7.72)	2184 (8.16)	1.06 (0.99; 1.12)	0.060	0.41	0.61
Pregnancy-related mortality, n, per 100000 live	50 (16.64)	129 (48.53)	2.92 (2.10; 4.04)	<0.001	31.89	32.88
Diabetes during pregnancy, n (%)	2634 (0.88)	2873 (1.09)	1.23 (1.17; 1.30)	<0.001	0.21	0.23
Hypertensive disorders of pregnancy, n (%)	11332 (3.80)	11331 (4.29)	1.13 (1.11; 1.17)	<0.001	0.47	0.58
Severe pre-eclampsia, n (%)	1630 (0.55)	1687 (0.64)	1.17 (1.09; 1.25)	<0.001	0.09	0.1
Postpartum haemorrhage (PPH), n (%)	2260 (0.76)	2292 (0.87)	1.15 (1.08; 1.22)	<0.001	0.11	0.13
Severe PPH, n (%)	1070 (0.36)	1145 (0.43)	1.21 (1.11; 1.31)	<0.001	0.07	0.083

Notes: [a]Denominators (number of deliveries: 258795, and livebirths: 260502) for 2021 were increased from 98% to 100%, to approximate full population coverage. [b]Total births were calculated as the sum of reported stillbirths and adjusted livebirths. ARDs - absolute risk differences.
