# Supplementary material for: Maternal and perinatal outcomes during successive and overlapping crises in Ukraine, 2019–2024: a nationwide population-based ecological study
Source: Lancet Reg Health Eur. 2026 Jul 14;68:101774. doi: 10.1016/j.lanepe.2026.101774 (PMC13382322; doi:10.1016/j.lanepe.2026.101774)
Supplement: Translated Abstract [file mmc5.pdf]

Резюме

Передумови

Україна зазнала взаємопов’язаних криз, спричинених пандемією COVID-19 та повномасштабною війною, що потенційно вплинуло на материнське та перинатальне здоров'я. Ми дослідили зміни відповідних показників на національному рівні між 2019 та 2024 роками.

Методи

У цьому популяційному екологічному дослідженні проаналізовано загальнодоступні дані Міністерства охорони здоров'я для вивчення тенденцій щодо кількості пологів і народжень, а також показників материнського та перинатального здоров'я. Зміни оцінювали за допомогою попарних порівнянь, абсолютних відмінностей ризиків та розмірів ефекту. Річні показники порівнювали з допандемічними значеннями, показниками попередніх років та періоду війни.

Результати

Кількість пологів та народжених дітей зменшилась приблизно на 40%, переважно протягом першого воєнного року. Між 2019 та 2024 роками (298066 та 176842 пологів відповідно) поширеність діабету під час вагітності зросла від 0,88% (n=2634) до 2,66% (n=4707), гіпертонічних розладів — від 3,80% (n=11332) до 5,45% (n=9633), тяжкої післяпологової кровотечі — від 0,36% (n=1070) до 0,53% (n=941). Серед 302190 та 179192 народжень відповідно, кількість передчасних нарождень збільшилась від 5,59% (n=16907) до 6,25% (n=11195), дітей з дуже малою масою при народженні — від 1,05% (n=3184) до 1,29% (n=2305), а з надзвичайно малою масою при народженні — від 0,44% (n=1335) до 0,60% (n=1068). Смертність, пов'язана з вагітністю, та перинатальна смертність, особливо мертвонародження, були вищими протягом другого року пандемії, але не під час війни. Рівень ранньої неонатальної смертності протягом усього періоду істотно не змінювався.

Інтерпретація

Поєднання кількох криз асоціювалося зі зниженням народжуваності та зростанням материнської захворюваності. Хоча під час пандемії смертність, пов'язана з вагітністю, та перинатальна смертність зросли, їхня відносна стабільність під час війни може свідчити про збереження медичних послуг для матерів і новонароджених, що, ймовірно, стало можливим завдяки міжнародній допомозі. Постійна підтримка спроможності служб охорони материнства та дитинства залишається пріоритетом у кризових умовах.

Фінансування

Це дослідження не отримувало фінансування.

“This translation in Ukrainian was submitted by the authors and we reproduce it as supplied. It has not been peer reviewed. Our editorial processes have only been applied to the original abstract in English, which should serve as reference for this manuscript.”
